# Characterizing Infrared Spectra of OH^–^·(H_2_O)_2_ and OH^–^·(H_2_O)_3_ with Constrained Nuclear-Electronic Orbital Molecular Dynamics

**DOI:** 10.1021/acs.jpca.5c04334

**Published:** 2025-10-18

**Authors:** Zhe Liu, Yiwen Wang, Yuzhe Zhang, Nan Yang, Yang Yang

**Affiliations:** Theoretical Chemistry Institute and Department of Chemistry, 5228 University of Wisconsin-Madison, 1101 University Avenue, Madison, Wisconsin 53706, United States

## Abstract

The vibrational spectra of OH^–^·(H_2_O)_*n*_ clusters for small *n* have been well established experimentally, with fundamental modes largely assigned. However, clear assignment of highly anharmonic modes and combination bands associated with strong hydrogen bonds, which often manifest as broad spectral features, remains challenging. In this work, we employ constrained nuclear-electronic orbital molecular dynamics (CNEO-MD) to provide detailed peak assignments and plausible physical interpretations for the vibrational spectra of OH^–^·(H_2_O)_n_ clusters with *n* = 2 and 3. The CNEO framework incorporates nuclear quantum effects, particularly nuclear quantum delocalization, through the underlying effective potential energy surfaces. When combined with classical molecular dynamics, CNEO-MD further captures coupling effects between vibrational modes. Leveraging machine-learned potentials, we perform a series of temperature-dependent CNEO-MD simulations and use the resulting spectra to facilitate peak assignment. Our results largely confirm the experimental assignments reported by Johnson and coworkers [*J. Chem. Phys.* **2016**, 145, 134304], while also providing direct, physically grounded interpretations of previously unassigned features.

## Graphical abstract

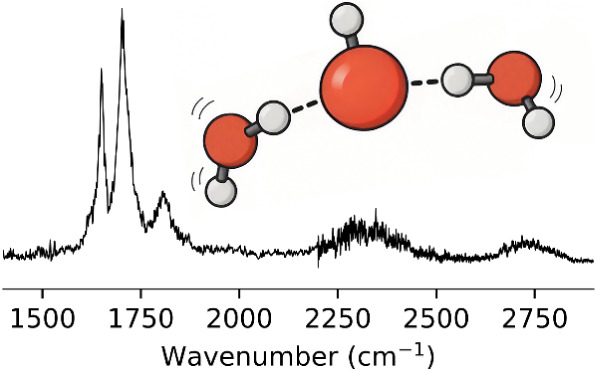

## 1 Introduction

The hydroxide ion (OH^–^) is a fundamental species in aqueous chemistry. It plays a central role in acid–base equilibria, proton transfer, and hydrogen-bonding networks.^1–3^ Gaining a molecular-level understanding of its interactions with water is essential for elucidating the microscopic mechanisms that govern these ubiquitous processes. In particular, small hydrated clusters, such as OH^–^·(H_2_O), OH^–^·(H_2_O)_2_, and OH^–^·(H_2_O)_3_ serve as ideal model systems for probing the structure and dynamics of OH^–^ solvation and hydrogen bonding.^1,4–10^

A key vibrational feature of hydroxide hydration clusters is the OH stretch of the water molecules hydrogen-bonded to the hydroxide ion, commonly referred to as ionic hydrogen bond (IHB) stretches.^7,9–11^ In the smallest cluster, OH^–^·(H_2_O), the vibrational zero-point energy (ZPE) exceeds the barrier for proton transfer, yielding an equilibrium structure in which a proton is symmetrically shared between two oxygen atoms.^5,12–16^ Adding a second water molecule to form OH^–^·(H_2_O)_2_, and then a third to form OH^–^·(H_2_O)_3_, shifts the shared proton away from the hydroxide, resulting in structures where OH^–^ is solvated by two or more water molecules connected through Figure 1 hydrogen bonds (Figure 1).^7,9,10^

**Figure 1:**
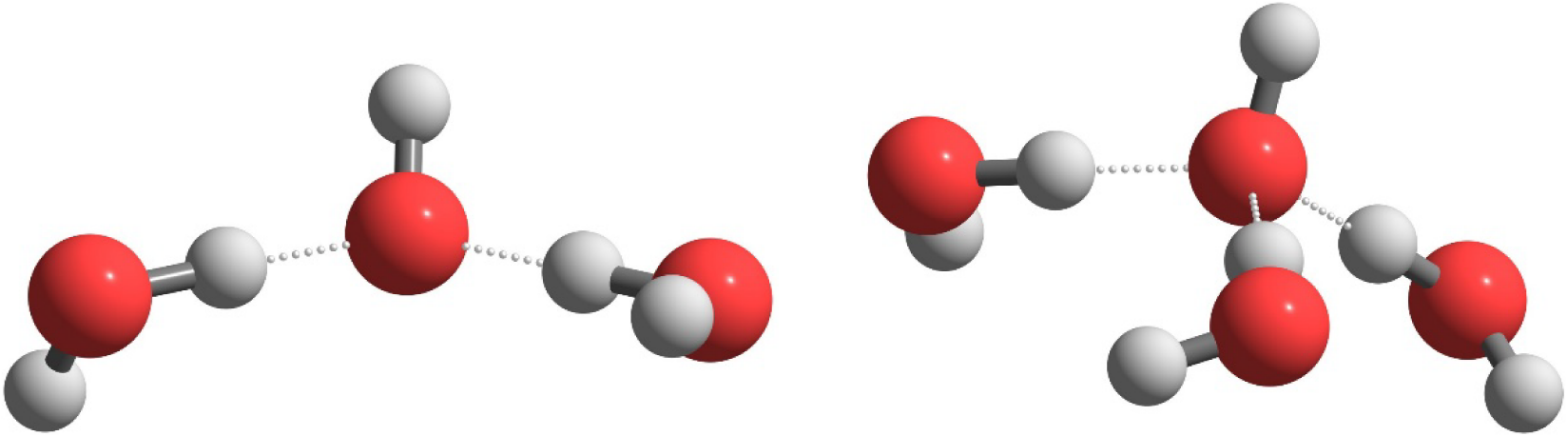
Structure of OH^–^·(H_2_O)_2_ and OH^–^·(H_2_O)_3_.

Despite their fundamental importance, detailed molecular insight into the vibrational spectra of OH^–^·(H_2_O)_2_ and OH^–^·(H_2_O)_3_ remains challenging to obtain through both experimental and theoretical approaches. Experimental studies by Johnson and coworkers, using H_2_-tagging vibrational spectroscopy, identified the fundamental HOH bending modes and some IHB stretching modes.^10^ However, the assignment of the symmetric IHB stretch mode in OH^–^·(H_2_O)_2_ remains ambiguous, and several strong spectral features in both clusters are still unexplained. Takahashi and coworkers^7,9,14,17^ predicted the vibrational spectra of these clusters with the multidimensional discrete-variable representation (DVR) method, including up to six normal modes for *n* = 2 and three local modes for *n* = 3. These studies accounted for anharmonicity and mode coupling and successfully reproduced some key spectral features, although the overall agreement with experiment remained limited. Johnson, McCoy, Jordan, and coworkers applied vibrational second-order perturbation theory (VPT2)^10^ to aid in peak assignments, but they noted that the large anharmonic shifts cast doubt on the reliability of perturbative treatments for these highly anharmonic systems. These challenges arise from the highly dynamic and fluxional nature of the hydrogen-bonded structures in OH^–^·(H_2_O)_n_ clusters, where pronounced zero-point energy effects, strong anharmonicity, and substantial mode coupling effects complicate both experimental interpretation and theoretical modeling.^5,7,9,12,14–16^

Recently, the constrained nuclear-electronic orbital (CNEO) theory was developed in our group to efficiently incorporate nuclear quantum effects, particularly quantum nuclear delocalization, into quantum chemistry calculations and molecular simulations.^18–20^ As part of the broader class of multicomponent quantum chemistry methods, CNEO treats both electrons and some or all nuclei quantum-mechanically. Unlike conventional multicomponent approaches, however, CNEO imposes a constraint on the expectation values of quantum nuclear position operators, resulting in an effective potential energy surface (PES) that depends on these expectation values along with the positions of classical nuclei.^18–20^ Molecular dynamics simulations on this CNEO effective PES—referred to as CNEO-MD—naturally include zero-point energy effects.^20,21^ Compared to conventional DFT-based *ab initio* molecular dynamics (AIMD), CNEO-MD incurs only a small increase in computational cost (typically less than 15%) when using CNEO density functional theory (CNEO–DFT).^16^

Previous studies have shown that CNEO–DFT and CNEO-MD significantly outperform conventional DFT-based methods in predicting hydrogen-related vibrational frequencies,^16,20–26^ proton distributions,^16,27–29^ reaction barriers and rate constants,^30,31^ and nonadiabatic proton transmission probabilities.^32^ Notably, CNEO has been successfully applied to charged water clusters, accurately capturing the shared-proton character in OH^–^·(H_2_O)^16^ and significantly improving the predicted vibrational spectra of protonated water clusters such as H_5_O_2_^+^, H_7_O_3_^+^ and H_9_O_4_^+^.^24^

In this study, we apply CNEO-MD to the challenging OH^–^·(H_2_O)_2_ and OH^–^·(H_2_O)_3_ complexes to characterize their vibrational spectra and resolve previously unassigned features. Section 2 outlines the CNEO theoretical framework and describes a Filter–Reconstruction–Projection approach used for spectral analysis. Section 3 details the computational methods. Section 4 presents harmonic and temperature-dependent CNEO-MD spectra and assigned complex features. Concluding remarks are provided in Conclusions section.

## 2 Method

### 2.1 CNEO Framework

The CNEO framework builds upon multicomponent quantum theories,^33–36^ in which both electrons and nuclei are treated quantum-mechanically. In a typical multicomponent density functional theory, such as the nuclear–electronic orbital density functional theory (NEO–DFT),^36–38^ the ground-state energy is obtained by evaluating the energy functional at the ground-state electronic density *ρ* ^*e*^ and quantum nuclear densities {*ρ* ^*n*^}.

1 $$E_{\mathrm{g.s.}}=E\left[\rho^{e},\left\{\rho^{n}\right\}\right]$$

In this formulation, the ground-state densities and energy depend solely on the positions of the classical nuclei, not on the quantum nuclear positions. As a result, the conventional concepts of molecular geometry and potential energy surfaces (PES) become ill-defined. To investigate dynamical properties such as vibrational spectroscopy, more sophisticated treatments are required, including NEO–DFT­(V)^39,40^ and real-time electron–nuclear dynamics.^41,42^

The CNEO framework addresses the challenges by imposing constraints on the expectation values of the position operator for each quantum nucleus ⟨**r**_*I*_⟩ and enforcing them to correspond to classical molecular geometries:^18,19^

2 $$\left\langle r_{I}\right\rangle =R_{I’}\forall I$$

Here **R**_*I*_ denotes the designated classical position of the *I*-th quantum nucleus. This treatment is justified by the fact that, in most chemical and biological systems, nuclei are much more localized than electrons and can be treated as distinguishable particles, with their position expectation values serving as well-defined molecular geometries.

Within CNEO–DFT, the ground-state energy is obtained by self-consistently solving a set of coupled Kohn–Sham equations for both electrons and quantum nuclei, subject to constraints on the nuclear position expectation values. This yields the CNEO effective PES, which depends on both the classical nuclear positions and the expectation values of the quantum nuclear position operators. While this PES closely resembles the conventional Born–Oppenheimer PES, it includes a key distinction: because CNEO treats some or all nuclei quantum mechanically, the PES inherently incorporates zero-point effects of quantum-mechanically treated nuclei,^18–20^ which are essential for accurately describing vibrational spectra, particularly for modes involving hydrogen motion.

Based on the CNEO effective PES, CNEO-MD simulations can be performed. The equations of motion for quantum nuclei are^20,21^

3 $$\frac{d}{dt}\left\langle r\right\rangle =\frac{\left\langle p\right\rangle }{m}$$

4 $$\frac{d}{dt}\left\langle p\right\rangle =-\nabla_{\langle r\rangle }V^{\mathrm{CNEO}}\left(\left\langle r\right\rangle \right)$$

where ⟨**r**⟩ and ⟨**p**⟩ are expectation values of position and momentum operators of the quantum nuclei, respectively, and *V* ^CNEO^(⟨**r**⟩) is the CNEO PES. For classical nuclei, the equations take the same form, with ⟨**r**⟩ and ⟨**p**⟩ in eqs 3 and 4 replaced by the classical positions **r** and momenta **p**. CNEO-MD closely resembles conventional AIMD, where energies and forces are computed on-the-fly using DFT. The key distinction is that CNEO-MD replaces DFT with CNEO–DFT, thereby incorporating nuclear quantum effects while incurring only a small computational cost increase that is typically less than 15%.^16^

### 2.2 Vibrational Spectra Calculation within CNEO Framework

Within the CNEO framework, vibrational spectra can primarily be computed in two ways. First, at equilibrium geometries, vibrational frequencies and normal modes are obtained by diagonalizing the mass-weighted CNEO–DFT Hessian matrix, which is constructed with respect to the expectation values of quantum nuclear position operators and the positions of classical nuclei.^22^ This static approach corresponds to a harmonic approximation within the CNEO framework. However, because the quantum nuclear wave functions in CNEO are intrinsically delocalized, the resulting frequencies incorporate some anharmonic effects that are absent in conventional harmonic analysis. Previous studies have shown that frequencies obtained from the CNEO–DFT Hessian are significantly more accurate than those from standard DFT, particularly for modes involving hydrogen motion.^16,22,24^

While the CNEO–DFT harmonic analysis can provide more accurate vibrational frequencies than DFT, it still does not capture strong mode coupling effects. To address this limitation, CNEO-MD offers a dynamic alternative that inherently incorporates both quantum nuclear delocalization and mode coupling effects. Like conventional AIMD, CNEO-MD for vibrational spectra is grounded in the quantum–classical correspondence of autocorrelation functions within the harmonic oscillator model.^20,21,23^ Vibrational spectra are then extracted by performing Fourier transforms of velocity or dipole autocorrelation functions obtained from CNEO-MD trajectories. This time-correlation approach not only recovers vibrational frequencies and intensities but also accounts for line broadening and peak shifting due to mode coupling effects.^24^ In some cases, it even captures overtones, combination bands, and Fermi resonances.^21,24,43^ Collectively, these features enable CNEO-MD to more closely reproduce experimental vibrational spectra than static CNEO harmonic analysis.

A critical aspect of this MD-based approach is the role of simulation temperature. The CNEO framework itself incorporates nuclear ZPE into the effective PES. The MD simulation, in turn, serves as a tool to introduce anharmonic intermode coupling by exploring regions of the PES away from the minimum. Therefore, temperature in CNEO-MD should be interpreted as an empirical parameter that controls the extent of anharmonic intermode coupling rather than a direct match to experimental thermal conditions. As found in a prior work by our group,^24^ this strategy effectively incorporates ZPE (handled by CNEO) and intermode coupling (activated by moderate-temperature MD), and empirically, a simulation temperature of 300 K often works well to recover the coupling effects and provides spectra with a good match with the experiments.

### 2.3 Peak Assignment with CNEO-MD

The assignment of peaks corresponding to fundamental vibrational modes is relatively straightforward and can be achieved by examining the associated normal modes from harmonic analysis, which reveal the characteristic nuclear motions. In contrast, peaks that appear only in MD trajectories—arising from mode coupling effects—are more difficult to interpret, as they often correspond to overtones or combination bands. To address this challenge, we herein introduce a Filter–Reconstruction–Projection approach for analyzing and assigning these nonfundamental features.

In our CNEO-MD simulations using the energy equipartition setup (*NVE*-eqp scheme),^24^ we typically run multiple trajectories, as spectra from individual trajectories often vary in peak positions, intensities, and even the number of observable features. Nevertheless, statistical averaging over multiple trajectories generally yields physically meaningful results. The required number depends on the specific system, but previous studies suggest that 30–50 trajectories of 2 ps each are generally required.^24^ For the systems investigated in this work, we found that even at 20 K, the averaged spectra exhibit clear mode coupling effects, giving rise to nonfundamental features such as overtones and combination bands. These features may appear strongly in some trajectories but remain weak or entirely absent in others.

To investigate a specific nonfundamental feature, we first identify a low-temperature MD trajectory (20 K in this study) in which the feature appears prominently. We then apply Fourier-based frequency filtering individually to each of the 3*N* time-dependent Cartesian coordinates of the trajectories, where *N* is the number of atoms in the system, retaining only the components that fall within the frequency range of the target feature.

After filtering, only the components within the target frequency range remain. These are used to reconstruct a filtered trajectory that captures the vibrational motions contributing to the selected spectral region—this constitutes the reconstruction step.

The reconstructed trajectory can be directly visualized to identify dominant vibrational patterns. For more quantitative analysis, it is projected onto the harmonic normal modes, yielding time-dependent projection amplitudes that reflect the extent to which each mode contributes to the filtered motion. These contributions can be further quantified by computing the time variance of each mode’s projection amplitude, which is crudely related to its vibrational energy and, therefore, its contribution to the spectral feature.

Typically, nonfundamental peaks are more prominent when they lie close in frequency to fundamental modes, as they can resonate with and borrow intensity from nearby fundamentals. Consequently, the strongest projection often corresponds to an adjacent fundamental mode. However, to uncover the intrinsic nature of these nonfundamental features prior to resonance, we deliberately ignore the most intense contributors and instead focus on the next most significant modes. The frequencies of these modes often approximate the target feature through linear combinations (suggesting combination bands) or integer multiples (indicating overtones), providing insight into the feature’s physical origin.

We acknowledge that the entire Filter-Reconstruction-Projection process is performed in Cartesian coordinates. This choice can, in principle, misrepresent contributions from large-amplitude curvilinear motions, such as internal rotations, for which a curvilinear or internal-coordinate framework would be more rigorous. Fortunately, in the present systems simulated at 20 K, internal water rotations are hindered. These low-amplitude motions can therefore be reasonably described by normal modes.

A detailed application of the Filter–Reconstruction–Projection approach is presented later for the OH^–^·(H_2_O)_2_ and OH^–^·(H_2_O)_3_ clusters, which serve both as illustrative examples and as the primary targets of this study.

## 3 Computational Details

The choice of electronic exchange–correlation functional can significantly influence the accuracy of computed vibrational spectra. For CNEO to perform well, it is essential to use an electronic functional with relatively small intrinsic electronic error.^22^ In previous investigations of small water clusters, including OH^–^·(H_2_O), H_5_O_2_^+^, H_7_O_3_^+^ and H_9_O_4_^+^, the performance of several commonly used functionals was benchmarked against CCSD­(T) for both free and hydrogen-bonded O–H stretching modes.^16,21,24,44^ It was found that PBE0,^45,46^ ωB97X,^47^ and ωB97MV^48^ have consistently shown superior performance. In Table S1, we benchmark the harmonic frequencies of OH^–^·(H_2_O)_2_ computed with these functionals against CCSD­(T) calculations. They again show good performances. Although ωB97MV with the def2-TZVPPD basis set^49,50^ gives the best agreement with CCSD­(T), PBE0 with the same basis set is presented in the main text to keep consistency with the prior CNEO study on water clusters.^24^ The ωB97MV data, which lead to a similar peak assignments, are provided in the Supporting Information.

In our CNEO–DFT calculations, only the protons were treated quantum mechanically, though the CNEO framework in principle allows a full quantum treatment of all nuclei.^19^ For the quantum protons, we employed the PB4D nuclear basis set.^51^ No electron–proton correlation (epc) functional^52–54^ was used, as previous studies have found that existing epc functionals have negligible impact on vibrational spectra for systems of this type.^44^

Vibrational spectra were obtained by Fourier transforming the dipole derivative autocorrelation functions. A common approach for generating these functions involves first equilibrating the system using a canonical ensemble (*NVT*) simulation, followed by multiple microcanonical (*NVE*) simulations initiated from uncorrelated configurations sampled from the *NVT* trajectory.^43,55^ We refer to this procedure as the *NVT*–*NVE* scheme. In contrast, our group has previously introduced a direct microcanonical approach with energy equipartition presets, denoted as *NVE*-eqp.^24^ This method bypasses the extensive *NVT* sampling while still yielding accurate vibrational spectra, and all MD spectra presented in this work were generated using the *NVE*-eqp approach. In the *NVE*-eqp scheme, multiple simulations are initiated from the optimized geometry, with kinetic energy *E* = *k*_B_ *T* assigned to each normal mode and velocity directions randomly chosen. While spectra from individual trajectories often differ in peak positions, intensities, and even the number of observable features, averaging over 30–50 trajectories typically yields a statistically meaningful spectrum.^24^ However, by leveraging the efficiency of machine-learned potential energy surfaces, we were able to perform and average over 200 trajectories with 10 ps each to ensure higher spectral convergency in this work.

For the neural network potential (NNP) used in the MD simulations, the initial training data set was generated from 50 direct *NVE*-eqp CNEO-MD trajectories at 300 K, each 2 ps in length. Notably, these trajectories are already able to produce meaningful vibrational spectra. Still, they were used as the starting point for training the NNP. To effectively explore undersampled regions of configuration space, we employed adaptive sampling,^56,57^ in which two NNP models were trained concurrently from different random seeds, and discrepancies in their predicted forces were used to identify undersampled configurations. Specifically, during NNP-based MD simulations, both models evaluated the forces on each structure, and any configuration showing a force disagreement beyond a preset threshold was labeled as undersampled.^56^ For each undersampled configuration, a CNEO–DFT calculation was performed, and the resulting energy and forces were added to the training set to refine the NNP. A separate dipole moment model was trained using a similar adaptive procedure. Once trained, the final NNP was used to generate all spectra presented in this work. A total of 200 trajectories, each 10 ps in length, was found to be sufficient for obtaining well-converged spectra (see Figures S2 and S3 for convergence analysis).

Harmonic analyses were performed using a locally modified version of PySCF,^58,59^ available through our group’s GitHub repository.^60^ MD simulations were carried out using the Atomic Simulation Environment (ASE),^61^ and the NNPs were trained using the DeePMD-kit.^62,63^ The filtering step in the Filter–Reconstruction–Projection analysis was achieved using a bandpass filter from the *scipy.signal* package.^64^

## 4 Results and Discussions

### 4.1 OH^–^·(H_2_O)_2_

Figure 2a shows the experimental vibrational predissociation spectrum of the H_2_-tagged OH^–^·(H_2_O)_2_ cluster.^10^ Five major peaks are observed. The first three peaks (labeled a_1_-a_3_) are relatively sharp, with peak positions at 1669, 1730, 1819 cm^–1^, respectively. In contrast, the remaining two peaks (a_4_ and a_5_) are broader and centered at 2310 and 2731 cm^–1^, respectively. Johnson and coworkers unambiguously assigned peaks a_1_ and a_2_ to the HOH bending modes and the asymmetric OH IHB stretch, respectively. This assignment is supported by isotope-dependent vibrational scaling (H/D substitution) results as well as by comparison with the more clearly resolved F^–^·H_2_O complex. However, the nature of the remaining peaks (a_3_–a_5_) remains unresolved. Table 1 summarizes the experimental peak positions and available assignments, alongside results from several computational methods discussed below.

**Figure 2:**
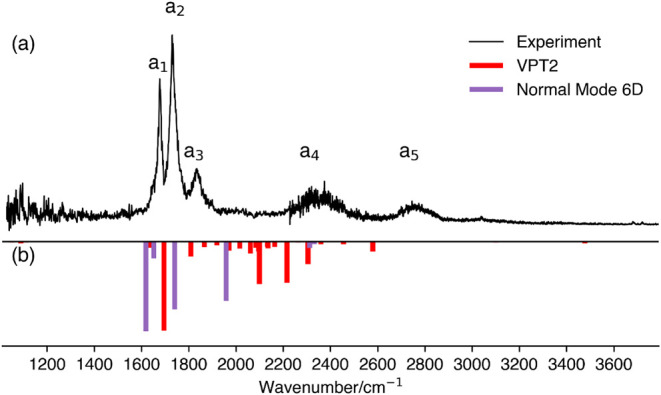
Vibrational spectra of OH^–^·(H_2_O)_2_ from (a) H_2_ tagging vibrational predissociation spectroscopy (ref 10) and (b) previous theoretical predictions from VPT2 (red; ref 10 and multidimensional DVR calculations using six normal modes (purple; ref 9).

**Table 1:** Experimental Vibrational Peak Positions (In cm^–1^) for OH^–^·(H_2_O)_2_, Compared with Theoretical Values from CNEO–DFT Harmonic Analysis, VPT2,^10^ and Multidimensional DVR Calculations Using Six Normal Modes (NM-6D).^a^ ^9^

Label	Expt.	VPT2	NM-6D	CNEO Harmonic	CNEO-MD 300 K	CNEO Assignment
a_1_	1669	1642	1651	1614	c_1_, 1635	c_1_, ${\mathrm{Bend}}_{sym}^{\mathrm{HOH}}$
		1638	1740	1627		c_1_, ${\mathrm{Bend}}_{asym}^{\mathrm{HOH}}$
a_2_	1730	1695	1618	1920	c_3_, 1820	c_3_, ${\mathrm{IHB}}_{asym}^{\mathrm{OH}}$
		2305	1958	2207		${\mathrm{IHB}}_{sym}^{\mathrm{OH}}$
a_3_	1819				c_2_, 1820 or c_4_, 2044	c_2_, Rock+Bend^OOP^ or c_4_, ${\mathrm{IHB}}_{sym}^{\mathrm{OH}}$
a_4_	2310				c_5_, 2213	c_5_, ${\mathrm{IHB}}_{sym}^{\mathrm{OH}}$
a_5_	2731				c_6_, 2447	c_6_, 2 $\nu_{\mathrm{Bend}}^{\mathrm{OOP}}$ , ${\mathrm{IHB}}_{asym}^{\mathrm{OH}}$ +Rot^OH^, and Rock+Bend^HOH^

a Bend^OOP^ denotes out-of-plane bending of water molecules. Rot^OH^ denotes hindered rotation of the OH^–^ group.

Figure 2b shows previously computed results from multidimensional DVR calculations using six normal modes (labeled NM-6D) and from VPT2, presented as inverted bars in purple and red, respectively. In the NM-6D calculations from Takahashi and coworkers,^9^ four major peaks were identified: the most intense peak at 1618 cm^–1^ corresponds to the asymmetric OH IHB stretch; a weaker peak at 1651 cm^–1^ is assigned to the symmetric HOH bending mode; a third peak at 1740 cm^–1^ is attributed to the asymmetric HOH bending mode; and a fourth peak at 1958 cm^–1^ is assigned to the symmetric OH IHB stretch. Additionally, weak features near 2300 cm^–1^ and 2900 cm^–1^ are attributed to combination bands and overtones. While the computed peak positions are in moderate agreement with experiment, the specific assignments raise some concerns. In particular, the asymmetric OH IHB stretch appears at a lower frequency than the bending modes, which contradicts experimental trends and challenges the physical plausibility of the assignment.

The VPT2 calculations performed by Johnson, McCoy, Jordan, and coworkers^10^ yielded many more vibrational features. The bending modes appearing at 1638 and 1642 cm^–1^ are in great agreement with the experimental a_1_ peak. The most intense feature was located at 1695 cm^–1^ and assigned to the asymmetric OH IHB stretch, closely matching the experimental a_2_ peak. However, this mode exhibited a substantial anharmonic correction—shifting by approximately 800 cm^–1^ from the harmonic frequency. Due to this unusually large shift, the authors expressed caution regarding the reliability of the VPT2 results for this system and included these assignments only in their Supplementary Material.

Our CNEO–DFT harmonic analysis results are shown in Figure 3 as blue bars. In the spectral region between 1200 and 3000 cm^–1^, four normal modes were identified with frequencies at 1614, 1627, 1920, and 2207 cm^–1^. The first two correspond to the symmetric and asymmetric HOH bending modes, separated by only 13 cm^–1^ and thus difficult to distinguish in the plot. The peak at 1920 cm^–1^, which exhibits the highest intensity, corresponds to the asymmetric OH IHB stretch. The higher-frequency peak at 2207 cm^–1^ is attributed to the symmetric OH IHB stretch. The splitting between these two IHB stretching modes (∼300 cm^–1^) is significantly larger than that between the bending modes. While the bending mode frequencies agree well with the experimental a_1_ peak, the predicted positions of the stretching modes do not directly match any of the remaining experimental features, leaving the assignments for a_2_–a_4_ ambiguous based on the CNEO harmonic analysis alone.

**Figure 3:**
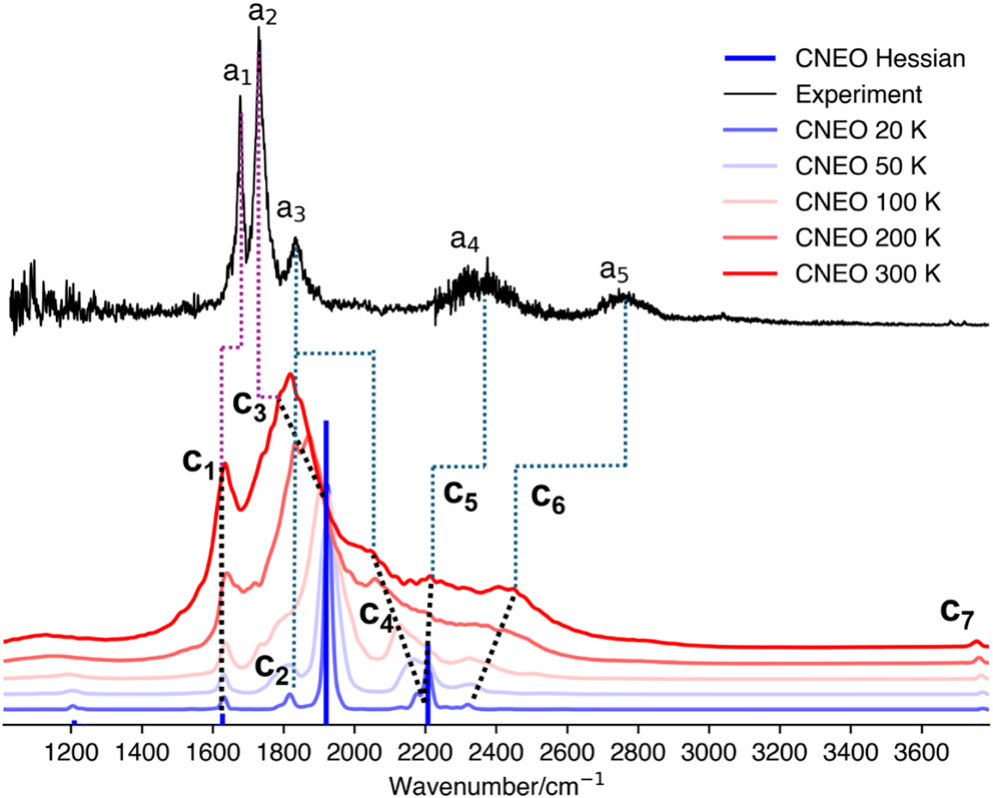
IR spectra of OH^–^·(H_2_O)_2_ obtained from CNEO–DFT harmonic analysis (blue bars) and CNEO-MD simulations at different temperatures (20, 50, 100, 200, and 300 K), compared with the experimental vibrational predissociation spectrum of the H_2_-tagged OH^–^·(H_2_O)_2_ cluster.^10^ Black dotted lines indicate the progression of CNEO-MD peaks with increased temperature. Purple dotted lines mark experimentally known peaks with assignments consistent with this work, while blue dotted lines highlight previously unassigned experimental features for which new assignments are proposed here.

Next, we carry out CNEO-MD simulations to incorporate mode coupling effects. The resulting spectra at various MD temperatures are shown in Figure 3. At 20 K, the CNEO-MD spectrum closely resembles the CNEO–DFT harmonic result. A small peak appears near 1635 cm^–1^ (c_1_), corresponding to the symmetric and asymmetric HOH bending modes. The most intense feature, at 1820 cm^–1^ (c_3_), is assigned to the asymmetric OH IHB stretch, while a moderately intense peak just above 2200 cm^–1^ corresponds to the symmetric OH IHB stretch. In addition to these fundamental modes, a weak feature emerges near 2180 cm^–1^, partially overlapping with the symmetric stretch. Two additional weak peaks also appear near 1820 cm^–1^ (c_2_) and 2320 cm^–1^ (c_6_), neither of which are present in the harmonic spectrum.

As the simulation temperature increases, the bending mode peak 1635 cm^–1^ remains nearly constant in frequency, but its intensity increases more noticeably than that of the other peaks. In contrast, the asymmetric OH IHB stretch peak near 1820 cm^–1^ (c_3_) undergoes a significant redshift and progressive broadening. The varying directions of these thermal shifts are noteworthy. We guess that this behavior is related to the anharmonic shape of the effective PES and the nature of the mode couplings, though a definitive answer would require further investigations, which is beyond the scope of current study. While the harmonic analysis places this mode at 1920 cm^–1^, its position shifts to approximately 1820 cm^–1^ at 300 K, aligning more closely with the experimental a_2_ peak, which is known to correspond to this vibrational mode.

The symmetric OH IHB stretch also exhibits strong temperature dependence, along with the nearby feature at 2180 cm^–1^. As the temperature increases to 50 K, the two peaks show comparable intensity and begin to merge into a broad band. By 100 K, only a single peak that red-shifted to 2130 cm^–1^ remains, although a shoulder is still visible near 2200 cm^–1^. With further temperature increase, this peak continues to shift and broaden. Notably, the intensity across the entire 2000–2400 cm^–1^ region remains high, indicating enhanced mode coupling and substantial thermal broadening, which tends to exaggerate spectral widths at elevated temperatures. At 300 K, the main peak shifts to 2044 cm^–1^ (c_4_), accompanied by a smaller feature at 2213 cm^–1^ (c_5_). However, it is not clear which of these corresponds to the fundamental symmetric OH IHB stretch and which originates from the 2180 cm^–1^ feature observed at 20 K. Accordingly, in Figure 3, we assign the same starting point to the trend lines for both c_4_ and c_5_ to reflect this ambiguity.

Based on this behavior, one possible assignment is that the experimental a_3_ peak corresponds to the red-shifted 2044 cm^–1^ (c_4_) peak, while the a_4_ peak may arise from the 2213 cm^–1^ (c_5_) peak. However, an alternative assignment emerges when examining the evolution of the c_2_ peak at 1820 cm^–1^. From 20 to 50 K and then to 100 K, the position of this peak remains relatively unchanged, although it becomes increasingly obscured by the shoulder of the red-shifted c_3_ peak, which corresponds to the asymmetric OH IHB stretch. It is worth noting again that thermal broadening in MD spectra is often exaggerated relative to experimental observations. If the asymmetric OH IHB stretch shifts closer to the 1800 cm^–1^ region with a narrower line width than currently simulated, the a_3_ peak could remain at its experimental position and plausibly correspond to the 1820 cm^–1^ feature. Therefore, we consider both 1820 cm^–1^ (c_2_) and 2044 cm^–1^ (c_4_) as possible candidates for the a_3_ peak in the CNEO-MD spectra, leaving this assignment only partially resolved.

Regardless of the final assignment for the a_3_ peak, we propose that the a_4_ feature originates from the c_5_ peak. Although the c_5_ feature is not very prominent in the CNEO-MD spectra at 300 K due to thermal broadening and spectral overlap, and may even appear as noise, we emphasize that it is a genuine spectral feature, which is reproducible even when averaging over 1000 trajectories (Figure S3). Additionally, this feature is also observed using the ωB97MV functional (Figure S7) in CNEO-MD calculations. Furthermore, as previously benchmarked, the vibrational frequency of the symmetric OH IHB stretch is underestimated by approximately 180 cm^–1^ when using the PBE0 functional with the def2-TZVPPD basis set. Accounting for this electronic error could possibly shift the predicted positions of c_5_ peak closer to the experimental a_4_ peaks. Based on this assignment, the small but distinct c_6_ feature, which grows in intensity and exhibits a blueshift with increasing temperature, should correspond to a_5_.

To elucidate the physical origin of each peak, we applied the Filter–Reconstruction–Projection analysis described in the Methods section. The results are shown in Figure 4. For the 1820 cm^–1^ feature at 20 K (c_2_), the dominant contribution arises from the asymmetric OH IHB stretch, which acts as the primary source of borrowed intensity. In addition, the two nearby bending modes contribute significantly to this feature. More intriguingly, several lower-frequency modes—specifically at 507, 564, 617, 1209, and 1238 cm^–1^—also show substantial contributions. Notably, the mode combinations 564 + 1238 cm^–1^ and 617 + 1209 cm^–1^ fall very close to 1820 cm^–1^, suggesting that this peak likely originates from combination bands involving these mode pairs. While the projection variances and frequency summations serve as our primary evidence for peak interpretation, we also note some interesting behavior associated with the time-dependent projection amplitudes, which might offer corroborating, albeit less definitive, support. For example, as shown in Figure 4a, the amplitudes of the 564 cm^–1^ and 1238 cm^–1^ modes exhibit correlated fluctuations, suggesting some cooperative behavior. However, a rigorous physical interpretation of such temporal correlations requires further justification, therefore, we remain mostly relying on projection variances and frequency summations as the basis for our peak interpretation.

**Figure 4:**
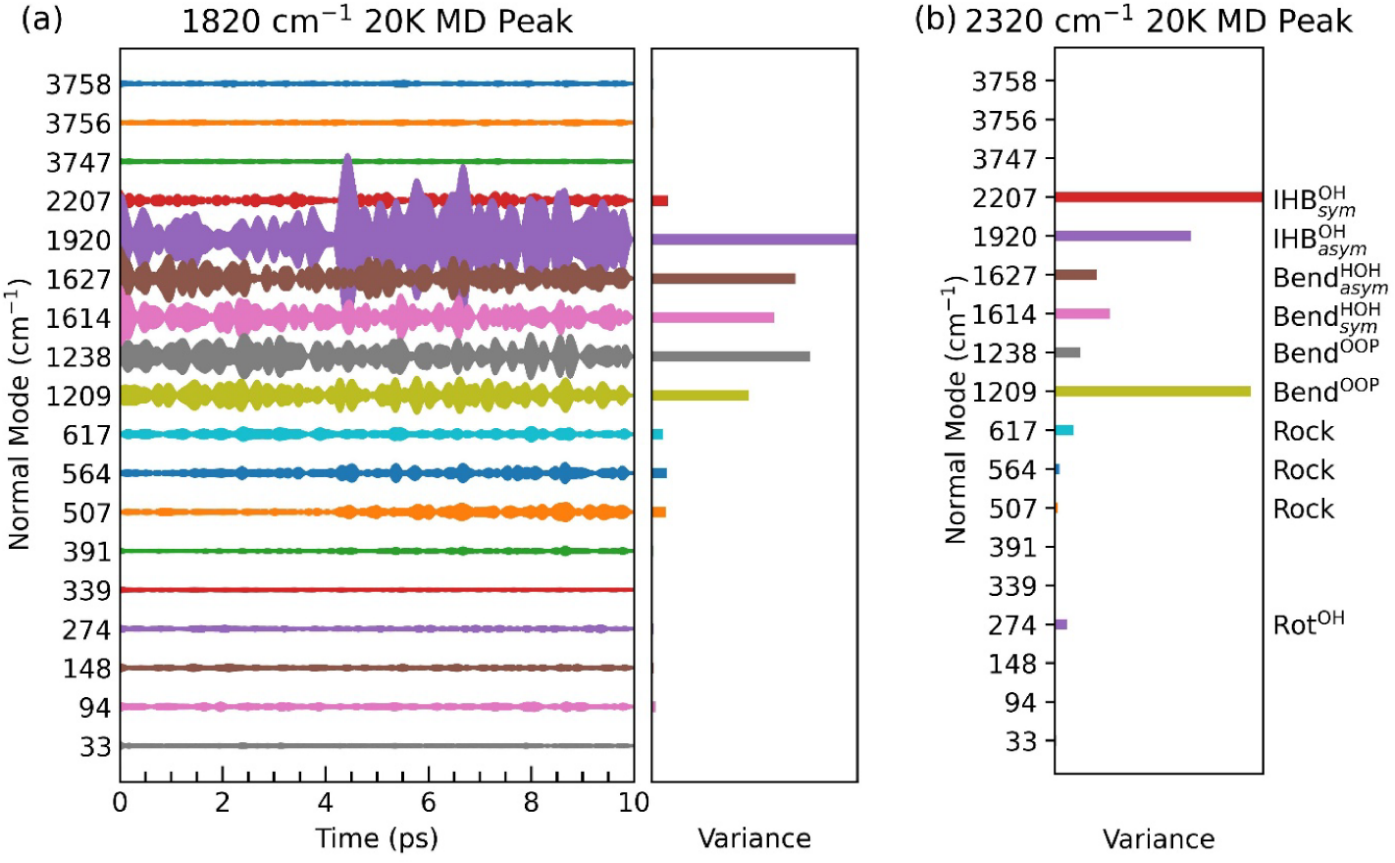
Positional projection amplitudes of a reconstructed CNEO-MD trajectory at 20 K onto harmonic normal modes, along with the variance of each projection amplitude over 10 ps, for the (a) 1820 cm^–1^ and (b) 2320 cm^–1^ peaks of OH^–^·(H_2_O)_2_. To highlight the contributions of less dominant modes, the variances of the strongest contributors, which are typically nearby fundamental modes that the peak resonates with, are capped at the right edge and not shown at full scale. Time-dependent amplitudes are provided in Figure S5.

Regarding the physical nature of the contributing modes, the lower-frequency modes (507–617 cm^–1^) correspond to rocking motions of the water molecules coupled with OH^–^ hindered rotation, while the 1209 and 1238 cm^–1^ modes are associated with out-of-plane bending motions of the water molecules.

We also performed the Filter–Reconstruction–Projection analysis for the 2320 cm^–1^ feature at 20 K (c_6_), which may correspond to the experimental a_5_ peak. The results indicate a more complex vibrational origin. The strongest contribution arises from the symmetric OH IHB stretch, serving as the main source of borrowed intensity. The second-largest contribution comes from the 1209 cm^–1^ out-of-plane bending mode, followed by the asymmetric OH IHB stretch at 1920 cm^–1^. In the low-frequency region, the 274 cm^–1^ mode—associated with hindered rotation of the OH^–^ unit—shows a much stronger contribution than its neighboring modes. Additional contributions are also observed from the 617 cm^–1^ rocking mode and the HOH bending modes near 1627 cm^–1^. Taken together, several mode combinations—including 2 × 1209 cm^–1^, 1920 + 274 cm^–1^, and 617 + 1627 cm^–1^—fall within the 2320 cm^–1^ region, suggesting that this feature originates primarily from a mixture of overtone and combination bands involving these modes.

In addition, ωB97MV calculations (Figures S7 and S8) yield temperature-dependent spectra that closely resemble PBE0 results while improving the agreement between c_5_ and experimental a_4_, owing to smaller deviations in OH IHB stretch frequencies. The dominant contributing modes to 20 K features remain the same (with only minor ordering differences), leading to identical physical assignments.

In summary, for the OH^–^·(H_2_O)_2_ cluster, the a_1_ and a_2_ peaks are further confirmed to correspond to the HOH bending modes (c_1_) and the asymmetric OH IHB stretch (c_3_), respectively. The previously unassigned a_4_ peak is now attributed to c_5_, which is closely related to the symmetric OH IHB stretch and its adjacent resonant feature. The a_5_ peak is assigned to c_6_, which arises from a combination of overtone and combination band contributions. The assignment of the a_3_ peak remains uncertain: it may correspond to the 1820 cm^–1^ feature (c_2_), which originates from combination bands involving rocking and out-of-plane bending motions of the water molecules, or alternatively, to c_4_, which is also related to the symmetric OH IHB stretch and its adjacent resonant feature but undergoes a pronounced redshift due to mode coupling effects.

### 4.2 OH^–^·(H_2_O)_3_

Figure 5a shows the vibrational predissociation spectrum of the H_2_-tagged OH^–^·(H_2_O)_3_ cluster. Four main features are observed. The first peak (a_1_), located at 1680 cm^–1^, is sharp and has been assigned to HOH bending modes. The second peak (a_2_) appearing at 1848 cm^–1^, is broader, and remains unassigned. The third feature consists of two smaller peaks, labeled $a_{3}^{\alpha }$ and $a_{3}^{\beta }$ , located at 2063 and 2140 cm^–1^, respectively; these are also unassigned. The most intense and broadest feature, labeled a_4_, peaks at 2586 cm^–1^ and has been attributed to overlapping contributions from asymmetric and symmetric OH IHB stretching modes. Table 2 summarizes the experimental peak positions and available assignments, alongside results from several computational methods discussed below.

**Figure 5:**
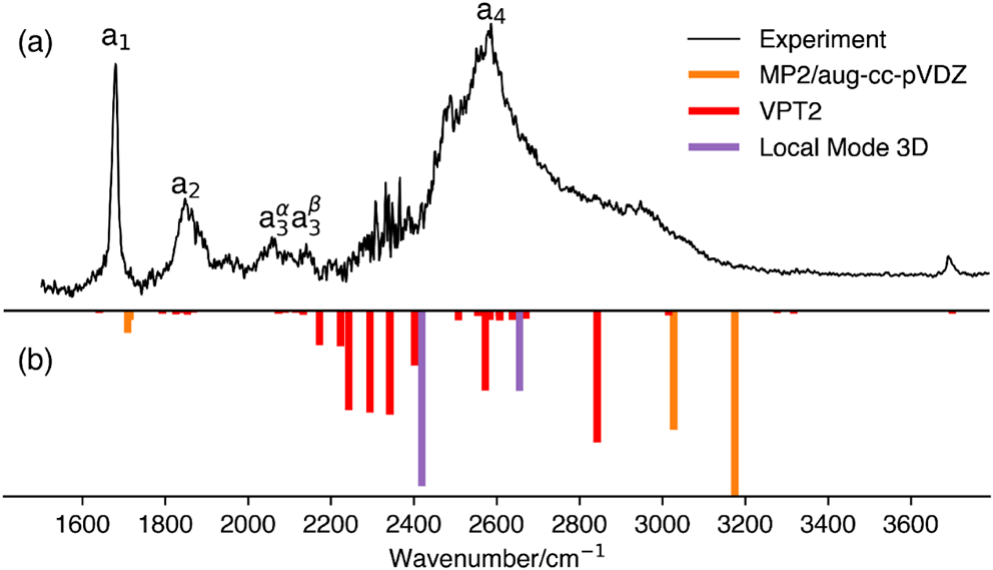
Vibrational spectra of OH^–^·(H_2_O)_3_ from (a) H_2_ tagging vibrational predissociation spectroscopy (ref 10) and (b) previous theoretical predictions from MP2/aug-cc-pVDZ harmonic analysis (orange; ref 10) VPT2 (red; ref 10) and multidimensional DVR calculations using three local modes (purple; ref 7) VPT2 HOH bending frequencies are not plotted due to unavailable intensity data.

**Table 2:** Experimental Vibrational Peak Positions (In cm^–1^) for OH^–^·(H_2_O)_3_, Compared with Theoretical Values from CNEO–DFT Harmonic Analysis, VPT2 (Ref 10), and Multidimensional DVR Calculations Using Three Local Modes (LM-3D, Ref 7)­^a^

Label	Expt.	VPT2	LM-3D	CNEO Harmonic	CNEO-MD 300 K	CNEO Assignment
a_1_	1680	1641, 1662		1638.5, 1638.7	c_1_, 1629	c_1_, Bend^HOH^
a_2_	1848				c_2_, 2014	c_2_, Rock+Bend^HOH^, 2 $\nu_{\mathrm{Bend}}^{\mathrm{OOP}}$
$a_{3}^{\alpha }$ , $a_{3}^{\beta }$	2063, 2140					
a_4_	2586	2402, 2342	2419, 2419	2470, 2471	c_4_, 2533	c_4_, ${\mathrm{IHB}}_{asym}^{\mathrm{OH}}$
a_4_ diffuse shoulder		2842	2665	2717		c_5_, ${\mathrm{IHB}}_{sym}^{\mathrm{OH}}$
						c_6_, Bend^OOP^+Bend^HOH^

a Bend^OOP^denotes out-of-plane bending of water molecules.

In Figure 5b, previously computed spectra from multidimensional DVR calculations using three local modes (LM-3D), from MP2/aug-cc-pVDZ harmonic analysis and from VPT2 are shown as inverted bars in purple, orange, and red, respectively. The LM-3D approach^7^ reports two main peaks: an intense feature at 2419 cm^–1^ assigned to the asymmetric OH IHB stretch and a weaker peak at 2665 cm^–1^ assigned to the symmetric OH IHB stretch. Although these assignments are physically reasonable, both peak positions deviate by over 150 cm^–1^ from the corresponding experimental features. Moreover, this approach offers no insight into the lower-frequency a_2_ and a_3_ peaks.

The VPT2 calculation^10^ yields many more features. It assigns the HOH bending modes to 1641 and 1662 cm^–1^, although these are not shown in Figure 5b due to unavailable intensity data.^10^ The asymmetric OH IHB stretches are predicted at 2342 and 2402 cm^–1^, while the symmetric stretch appears at 2842 cm^–1^. These assignments generally follow expected experimental trends. However, a notable concern is that the predicted intensities of the asymmetric stretches are lower than that of the symmetric stretch, which contradicts typical experimental observations. Additionally, several high-intensity peaks are found between 2200 and 2400 cm^–1^, but their correspondence to the experimental spectrum remains unclear—particularly regarding their possible relation to the unassigned a_2_ and a_3_ features.

Similar to the case of OH^–^·(H_2_O)_2_, we first performed a CNEO harmonic analysis for OH^–^·(H_2_O)_3_. The results, shown as blue bars in Figure 6, reveal three prominent features within the frequency range of interest. The first corresponds to a cluster of HOH bending modes located at 1605, 1638.5, and 1638.7 cm^–1^, in good agreement with the experimental a_1_ peak at 1680 cm^–1^. The second feature, appearing at 2470 and 2471 cm^–1^, arises from two nearly degenerate asymmetric OH IHB stretches and forms a strong composite peak that corresponds to the experimental a_4_ feature. The third peak, at 2717 cm^–1^, originates from the symmetric OH IHB stretch with moderate intensity and likely contributes to the diffuse shoulder observed experimentally near 2950 cm^–1^. While these harmonic predictions capture several key features, the a_2_ and a_3_ peaks remain unaccounted for.

**Figure 6:**
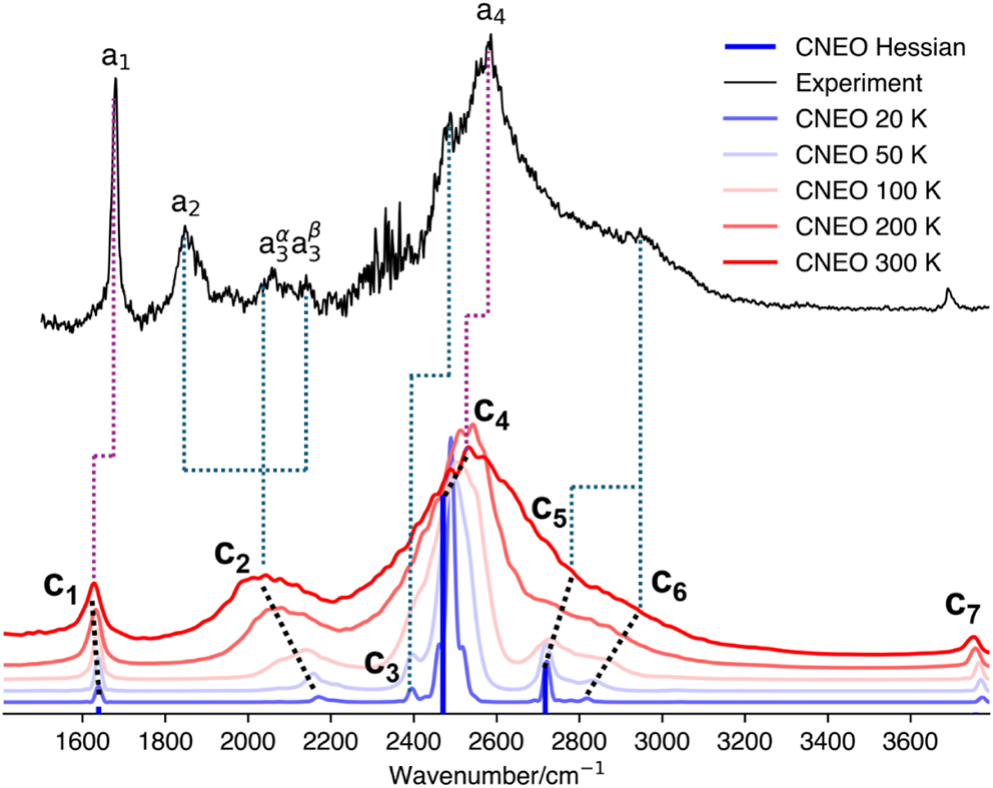
IR spectra of OH^–^·(H_2_O)_3_ obtained from CNEO–DFT harmonic analysis (blue bars) and CNEO-MD simulations at different temperatures (20, 50, 100, 200, and 300 K), compared with the experimental vibrational predissociation spectrum of the H_2_-tagged OH^–^·(H_2_O)_3_ cluster.^10^ Black dotted lines indicate the progression of CNEO-MD peaks with temperature. Purple dotted lines mark experimentally known peaks with assignments consistent with this work, while blue dotted lines highlight previously unassigned experimental features for which new assignments are proposed here.

We then performed CNEO-MD simulations for OH^–^·(H_2_O)_3_ at various temperatures, and the resulting spectra are shown in Figure 6. The HOH bending modes, labeled as c_1_, increase in intensity with rising temperature but remain nearly unchanged in frequency. The asymmetric OH IHB stretches (c_4_) exhibit a slight blueshift, improving agreement with the experimental a_4_ peak. The symmetric OH IHB stretch (c_5_) also blueshifts and undergoes significant broadening as the temperature increases. By 200 K, it appears as a shoulder on the c_4_ feature, and by 300 K, it becomes almost entirely obscured due to thermal broadening. As in the OH^–^·(H_2_O)_2_ case, this extent of broadening is likely overestimated due to the classical nature of the MD simulation, which tends to exaggerate temperature-induced spectral widths.

In addition to the fundamental modes, three notable peaks emerge in the 20 K CNEO-MD spectrum. The first appears at 2175 cm^–1^ (labeled c_2_) as a weak feature at low temperature but grows in intensity and broadens as the temperature increases. Its peak position also redshifts significantly, bringing it closer to the experimental a_2_ peak. Closer inspection of c_2_ reveals a main peak on the lower-frequency side and a diffuse shoulder on the higher-frequency side—closely resembling the combined shape of the experimental a_2_ and a_3_ features. This similarity suggests that the main peak corresponds to a_2_, while the shoulder may correspond to a_3_. However, due to the limited resolution and temperature broadening inherent to MD simulations, we are unable to resolve the fine structure of $a_{3}^{\alpha }$ and $a_{3}^{\beta }$ .

The second major nonfundamental peak appears at 2400 cm^–1^ (labeled c_3_). It is clearly visible at 20 and 50 K but becomes increasingly obscured by the broadening of the c_4_ feature as the temperature rises. Notably, the experimental spectrum shows a small peak near 2480 cm^–1^ on the lower-frequency side of a_4_. It is plausible that c_3_ corresponds to this experimental feature; however, due to the artificial broadening introduced by elevated temperatures, a definitive assignment remains challenging.

The third notable feature is a weak peak at 2820 cm^–1^ (labeled c_6_), which gradually increases in intensity and remains discernible up to 100 K. At higher temperatures, however, it merges with the symmetric OH IHB stretch (c_5_) and contributes to the broad shoulder on the high-frequency side of the a_4_ peak. Although this feature is not clearly resolved in the experimental spectrum, its temperature-dependent behavior and spectral position suggest that it influences the shape of the a_4_ band’s high-frequency tail.

We also investigated the physical origin of the three nonfundamental features using the Filter–Reconstruction–Projection analysis. Results are shown in Figure 7. For the c_2_ feature, which appears at 2175 cm^–1^ at 20 K, the largest contributions originate from the 2470, 2471, and 2717 cm^–1^ modes, indicating that this feature primarily borrows intensity from both the asymmetric and symmetric OH IHB stretches. Additional contributions are observed from the 1605 and 1639 cm^–1^ HOH bending modes. Notably, the 532 cm^–1^ mode, which is associated with the symmetric rocking motion of water molecules, contributes more significantly than its neighboring modes. When combined with the HOH bending frequencies, they yield a frequency sum that fall near 2175 cm^–1^, suggesting a combination band assignment involving water rocking and bending motions. Furthermore, substantial contributions from the 1082–1109 cm^–1^ out-of-plane bending modes suggest that overtones of these modes may also contribute. Taken together, the c_2_ feature likely arises from a mixture of overtone and combination band character. It is possible that a_2_ and a_3_ correspond to different contributions within this mixture; however, due to temperature broadening, the MD spectra lack the resolution necessary to unambiguously distinguish between them.

**Figure 7:**
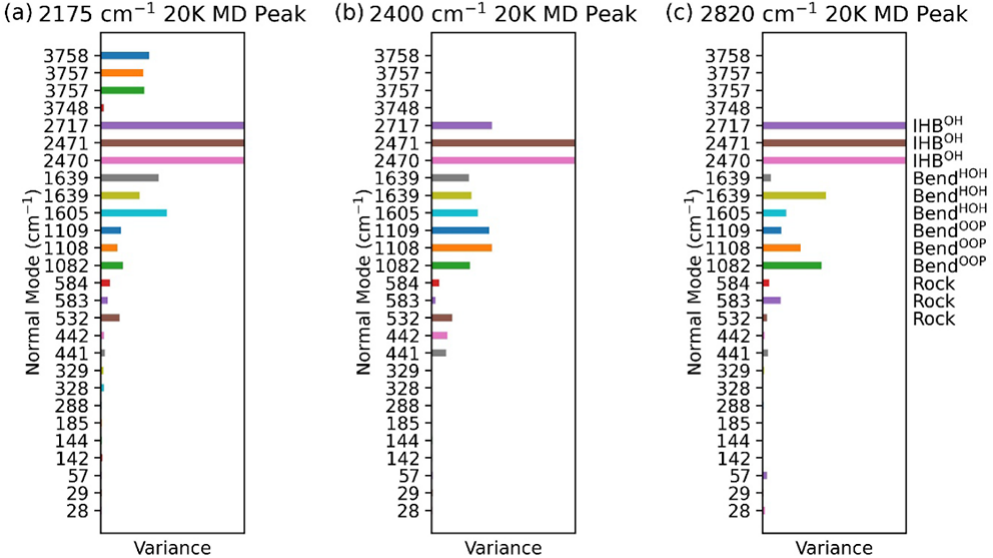
Variance of positional projection amplitudes of a reconstructed CNEO-MD trajectory at 20 K onto harmonic normal modes for the (a) 2175 cm^–1^, (b) 2400 cm^–1^, and (c) 2820 cm^–1^ peaks of OH^–^·(H_2_O)_3_. To highlight the contributions of less dominant modes, the variances of the strongest contributors, which are typically nearby fundamental modes that the peak resonates with, are capped at the right edge and not shown at full scale. Time-dependent amplitudes are provided in Figure S6.

For the c_3_ feature appearing at 2400 cm^–1^ peak at 20 K, the major contribution originates from the nearby asymmetric OH IHB stretch. In addition, significant contributions are observed from the 441, 442, and 532 cm^–1^ rocking modes coupled with OH^–^ hindered rotation, the ∼1100 cm^–1^ out-of-plane bending modes, and the ∼1600 cm^–1^ HOH bending modes. Among all the features examined in this study, this peak exhibits the most complex mixture of contributing modes. Ironically, despite the richness of these contributions, no single overtone or straightforward combination band clearly accounts for the 2400 cm^–1^ feature based on direct mode summation. This suggests that its origin may involve more intricate vibrational interactions, potentially including higher-order combinations or multimode coupling effects, such as a HOH bending mode combined with an overtone of water rocking motions.

For the c_6_ feature at 2820 cm^–1^ at 20 K, the primary contribution originates from the 2717 cm^–1^ symmetric OH IHB stretch, with additional significant input from the asymmetric stretch modes. Beyond these IHB stretching modes, notable contributions also arise from the ∼1100 cm^–1^ out-of-plane bending modes and the ∼1600 cm^–1^ HOH bending modes. Since the sum of these bending frequencies is close to 2820 cm^–1^, we assign this peak to a combination band involving both out-of-plane and in-plane bending motions of the water molecules.

In addition, ωB97MV calculations (Figures S9 and S10) reproduce the qualitative temperature evolution and overall feature mapping. All c_1_–c_6_ features are present with similar relative positions. Notably, with ωB97MV the c_2_ feature splits into two subpeaks, aligning well with the experimental bifurcation (a_2_ and a_3_) and lending further support to our peak assignment. At low temperature, the absence or weakness of c_2_ under ωB97MV may indicate a slightly higher energetic threshold for activating the combination band. The Filter–Reconstruction–Projection analysis with ωB97MV confirms the same qualitative modal origins for c_3_ and c_6_ as obtained with PBE0.

In summary, for the OH^–^·(H_2_O)_3_ cluster, the a_1_ and a_4_ peaks are confidently assigned to the HOH bending modes (c_1_) and the asymmetric OH IHB stretch (c_4_), respectively. The symmetric OH IHB stretch (c_5_) is evident in both the harmonic analysis and low-temperature MD spectra but becomes largely obscured within the broadened shoulder of the c_4_ feature at elevated temperatures. The previously unassigned a_2_ and a_3_ peaks are attributed to the c_2_ feature, which arises from a mixture of overtones and combination bands involving water rocking, in-plane bending, and out-of-plane bending modes coupled with the IHB stretches. However, due to temperature-induced spectral broadening, an unambiguous separation of the contributions to a_2_ and a_3_ remains challenging. The small peak on the low-frequency side of a_4_ may correspond to the c_3_ feature observed in the MD spectra, although its precise vibrational origin remains uncertain. Finally, the high-frequency shoulder on the right side of a_4_ is shaped not only by the symmetric OH IHB stretch (c_5_) but also by a combination band (c_6_) involving the ∼1100 and ∼1600 cm^–1^ bending modes.

## 5 Conclusions

In this work, we conducted a detailed computational investigation of the infrared vibrational spectra of small hydrated hydroxide clusters, specifically OH^–^·(H_2_O)_2_ and OH^–^·(H_2_O)_3_, using the constrained nuclear-electronic orbital molecular dynamics (CNEO-MD) approach. For both systems, CNEO-MD, assisted by temperature variation to modulate mode coupling, successfully captured most of the key experimental features. Our simulations further confirmed existing assignments for HOH bending modes and several IHB stretching modes. For previously unassigned experimental features, the combination of temperature-dependent dynamics and the Filter–Reconstruction–Projection technique enabled plausible physical interpretations, attributing them largely to overtones and combination bands.

From a theoretical perspective, this study underscores the importance of incorporating both nuclear quantum effects and mode coupling to accurately predict and interpret the vibrational spectra of strongly hydrogen-bonded systems. The CNEO potential energy surface inherently captures quantum nuclear delocalization, while the MD simulations introduce mode coupling effects that are critical for reproducing experimental spectra. Our results demonstrate that CNEO-MD, particularly when combined with machine-learned potentials, offers a powerful and computationally efficient framework for simulating complex vibrational dynamics. Although some assignments remain partially tentative, the present analysis narrows plausible interpretations and provides major contributing modes. These insights will benefit future higher-level anharmonic methods such as DVR that can now be guided by the reduced set of mode combinations. Future applications of this approach may extend to larger clusters, condensed-phase systems, and other environments where nuclear quantum effects play a central role in vibrational behavior and chemical reactivity.

## Supplementary Material





## Notes

The Neural Network Potential details; a comparison of DFT and CCSD­(T) frequencies (Table S1); a comparison between the *NVE*-eqp scheme and the *NVT*–*NVE* method (Figure S1); convergence tests as a function of the number of trajectories (Figures S2 and S3); power spectra before and after frequency filtering (Figure S4); PBE0 time-dependent normal modes projection amplitudes (Figures S5 and S6); ωB97MV spectra and normal modes projection amplitudes for OH^–^·(H_2_O)_2_ and OH^–^·(H_2_O)_3_ (Figures S7–S10) (PDF)
The CNEO harmonic frequencies and GIF animations of the vibrational modes (ZIP)
