# Supplementary material for: Characterizing Infrared Spectra of OH–·(H2O)2 and OH–·(H2O)3 with Constrained Nuclear-Electronic Orbital Molecular Dynamics
Source: J Phys Chem A. 2025 Oct 18;129(43):9883–94. doi: 10.1021/acs.jpca.5c04334 (PMC12581138; doi:10.1021/acs.jpca.5c04334)
Supplement: Supplementary file 1 [file jp5c04334_si_001.pdf]

# Supporting Information for:

# Characterizing Infrared Spectra of $OH^{-}\cdot(H_2O)_2$ and $OH^{-}\cdot(H_2O)_3$ with Constrained Nuclear-Electronic Orbital Molecular Dynamics

*Zhe Liu, Yiwen Wang, Yuzhe Zhang, Nan Yang, Yang Yang**

Theoretical Chemistry Institute and Department of Chemistry, University of Wisconsin-Madison, 1101 University Avenue, Madison, Wisconsin 53706, United States

Email: yyang222@wisc.edu

**Neural Network Potential**

The deep neural network potential was trained using the DeepMD-kit package.[1,2] The model maps the atomic coordinates and types to the total system energy. We employed the "se_e2_a" descriptor[3] and a fitting network with three hidden layers. The initial training set, comprising 200,000 samples from CNEO-MD simulations, was augmented with approximately 100,000 additional samples for each system via adaptive sampling. This dataset was randomly split into training, validation, and testing sets with an 8:1:1 ratio. The network was trained for 4,000,000 steps with an initial learning rate of 0.001. The final trained model achieved a mean absolute error (MAE) of 0.03 eV on the test set.

**Table S1** Harmonic frequencies (in $cm^{-1}$) of $OH^{-}\cdot(H_2O)_2$ computed with DFT, compared with CCSD(T) reference values.

| Method | CCSD(T) | PBE0 | ωB97X | ωB97MV |
|---|---|---|---|---|
| Basis | def2-TZVP | def2-TZVPPD | def2-TZVPPD | def2-TZVPPD |
| $\mathrm{Bend}_{sym}^{\mathrm{OH}}$ | 1740 | 1698 | 1709 | 1692 |
| $\mathrm{Bend}_{asym}^{\mathrm{OH}}$ | 1750 | 1706 | 1706 | 1698 |
| $\mathrm{IHB}_{asym}^{\mathrm{OH}}$ | 2467 | 2331 | 2587 | 2501 |
| $\mathrm{IHB}_{sym}^{\mathrm{OH}}$ | 2717 | 2540 | 2746 | 2672 |

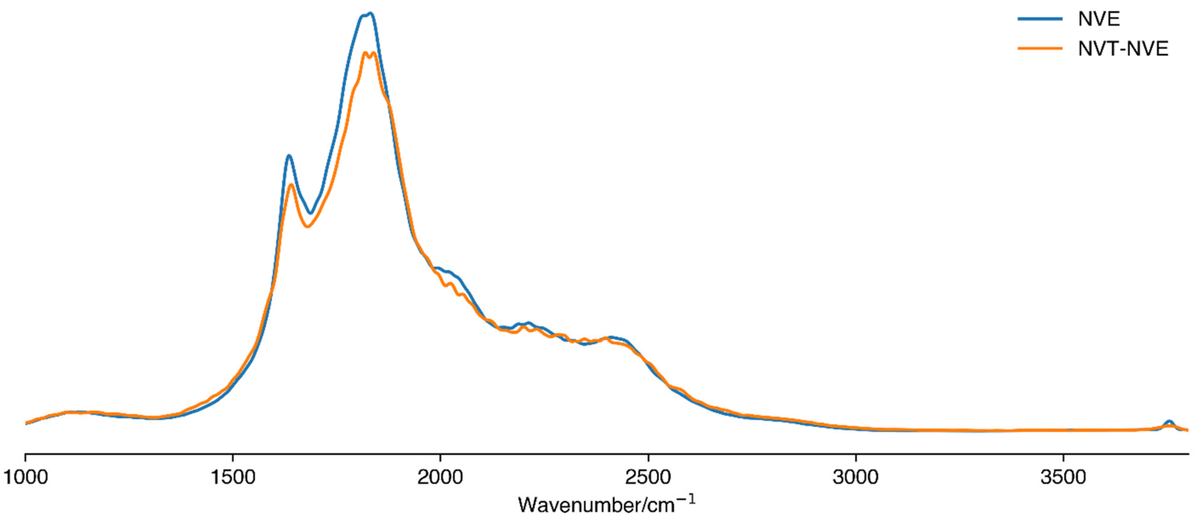


**Figure S1** CNEO-MD spectra of $OH^{-}\cdot(H_2O)_2$ comparing the *NVE*-eqp and *NVT–NVE* schemes for initial condition sampling. All simulations used machine-learned potentials. For the *NVE*-eqp scheme, 1000 *NVE* trajectories of 10 ps each were generated at 300 K. For the *NVT–NVE* scheme, a single 100 ps *NVT* trajectory was first generated, from which 1000 *NVE* simulations of 10 ps each were initiated using uncorrelated configurations sampled from the *NVT* trajectory.

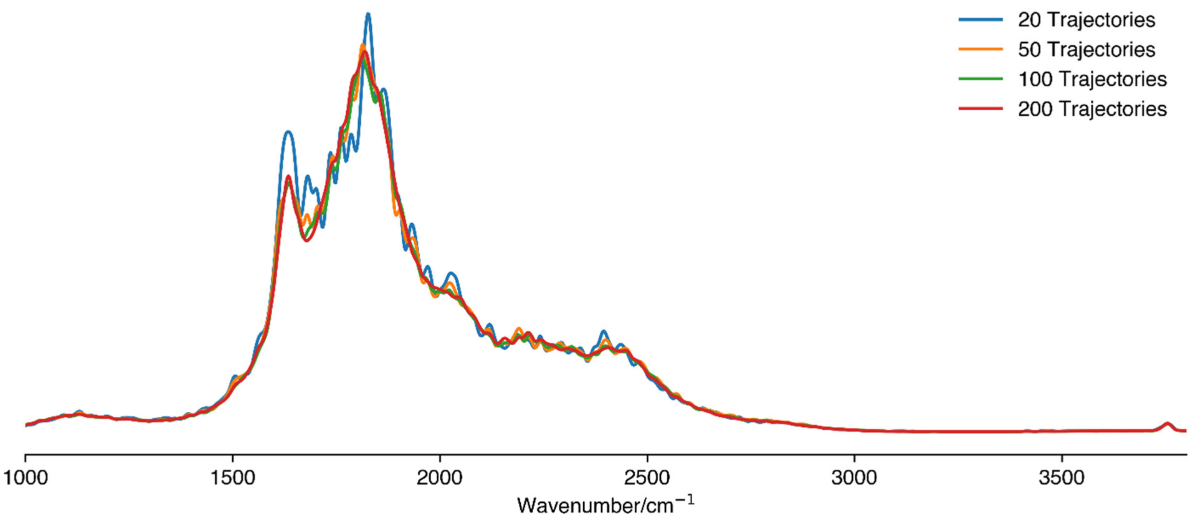

**Figure S2** CNEO-MD IR spectra of $OH^{-}\cdot(H_2O)_2$ as a function of the number of *NVE* trajectories (20–200), with each trajectory being 10 ps in length. Averaging over 100–200 trajectories is sufficient to achieve spectral convergence.

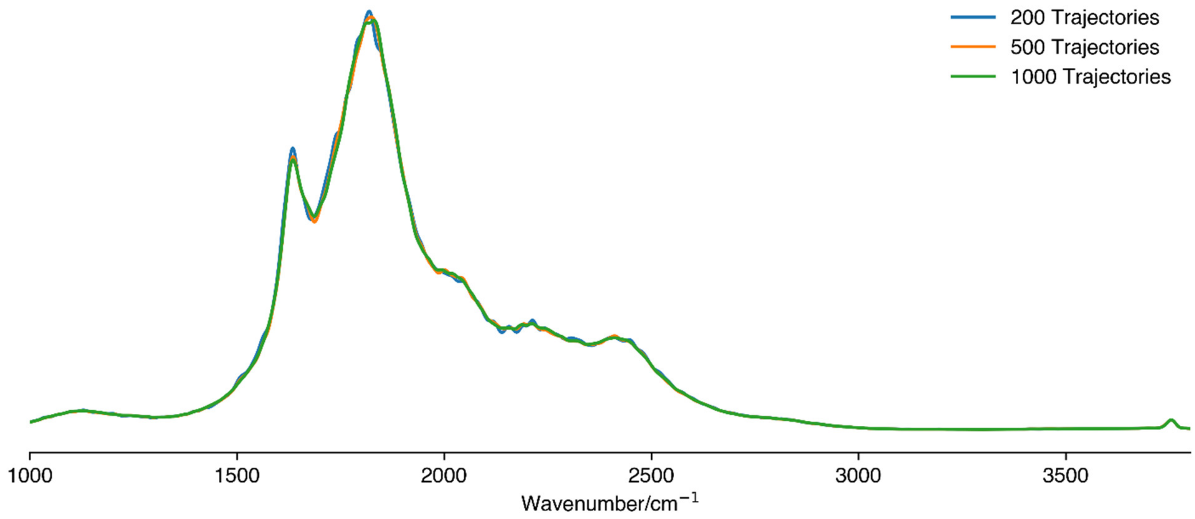


**Figure S3** CNEO-MD IR spectra of $OH^{-}\cdot(H_2O)_2$ as a function of the number of *NVE* trajectories (200–1000), with each trajectory being 10 ps in length. The spectra exhibit negligible differences once the number of trajectories exceeds 200.

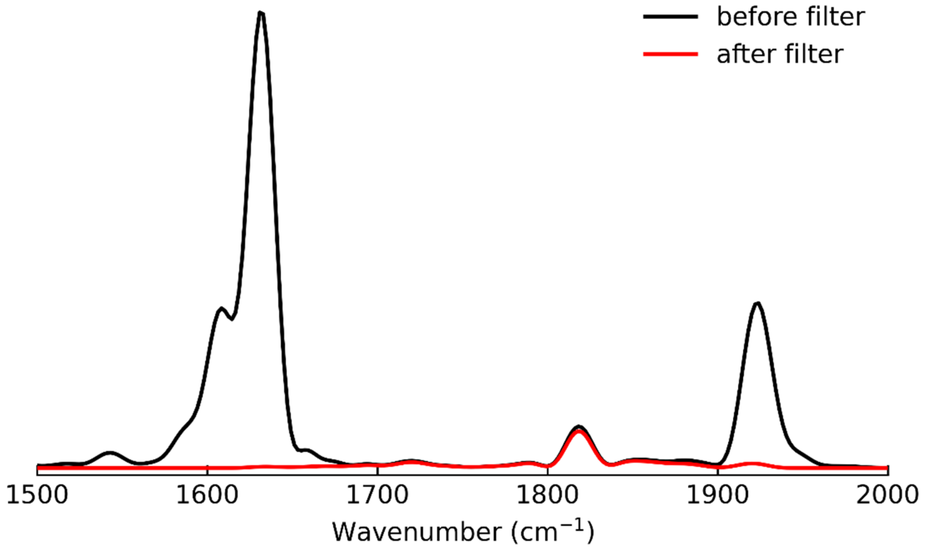


**Figure S4** Power spectra of $OH^{-}\cdot(H_2O)_2$ from a single trajectory a with strong feature at 1820 $cm^{-1}$, shown before and after applying the frequency filter. The filter effectively removes frequencies outside the targeted 1820 $cm^{-1}$ peak region.

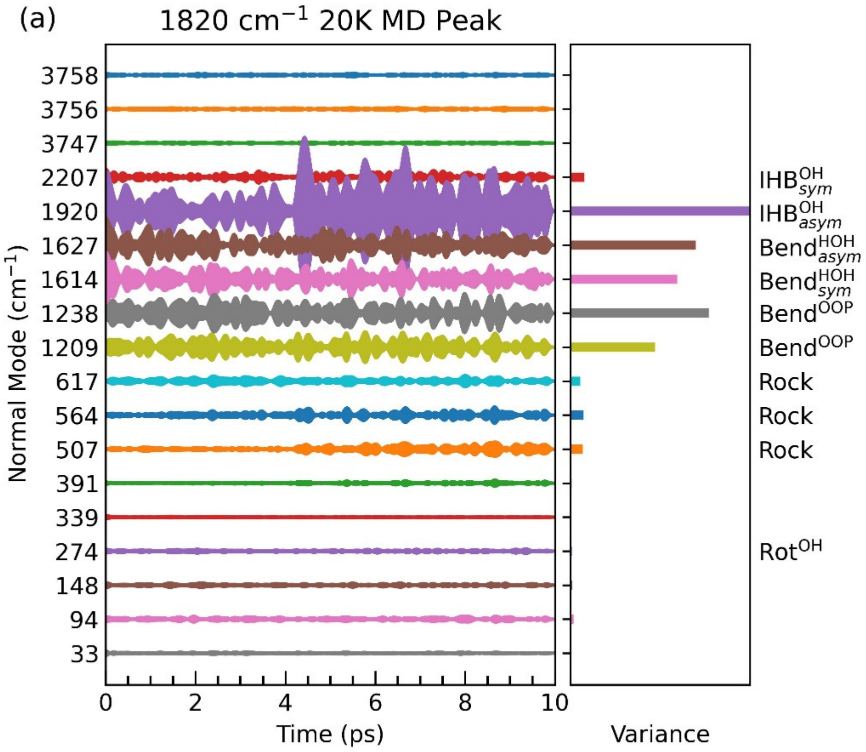

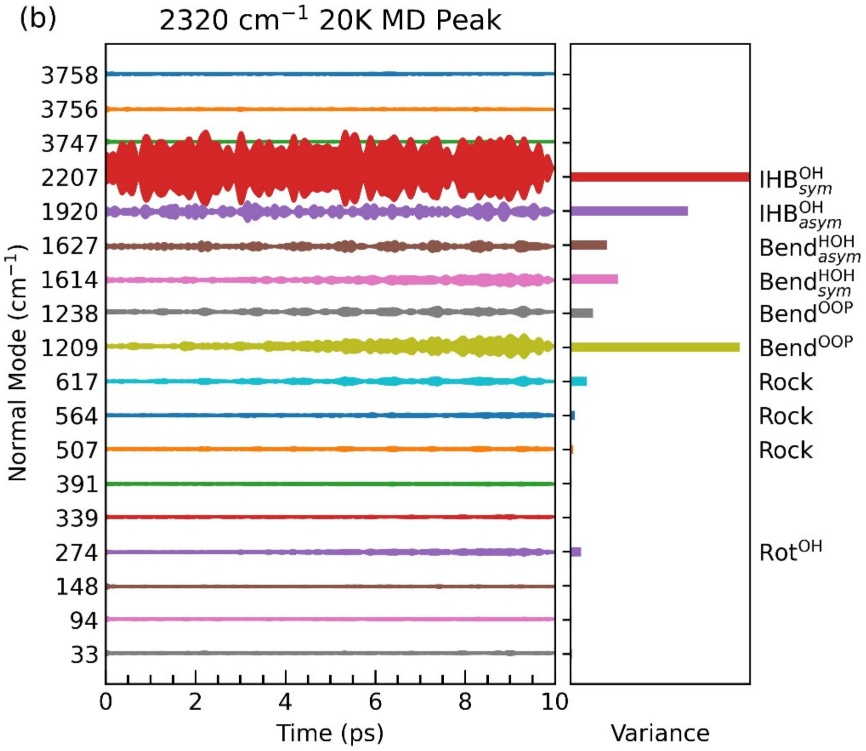


**Figure S5** Positional projection amplitudes of a reconstructed PBE0 CNEO-MD trajectory at 20K onto harmonic normal modes, along with the variance of each projection amplitude over 10 ps, for the (a) 1820 $cm^{-1}$ and (b) 2320 $cm^{-1}$ peaks of $OH^{-}\cdot(H_2O)_2$. To highlight the contributions of less dominant modes, the variances of the strongest contributors, which are typically nearby fundamental modes that the peak resonates with, are capped at the right edge and not shown at full scale.

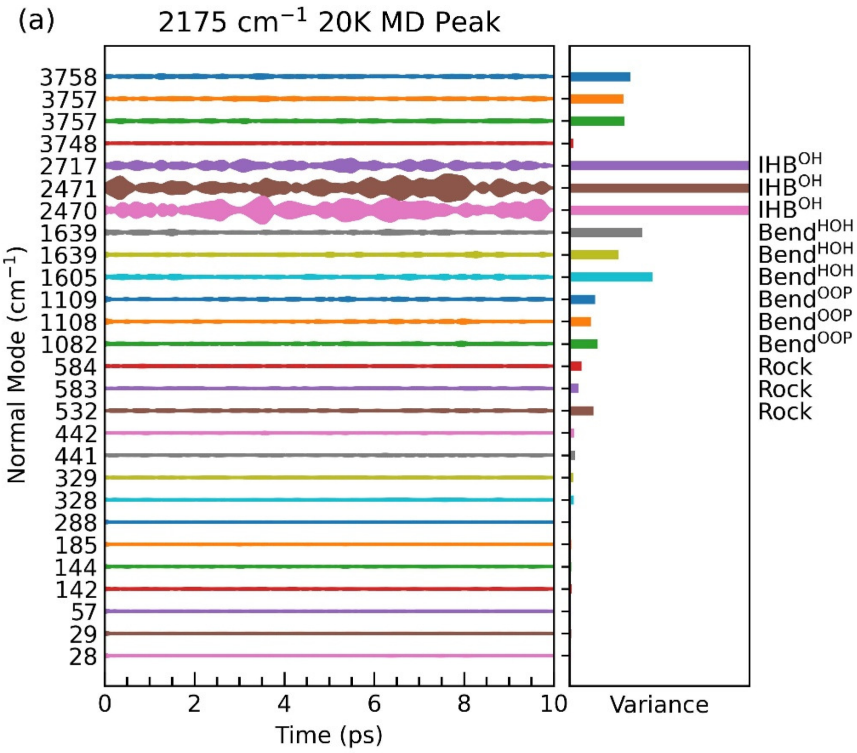
(a)
2175 cm$^{-1}$ 20K MD Peak
3758
3757
3757
3748
2717
2471
2470
1639
1639
1605
1109
1108
1082
584
583
532
442
441
329
328
288
185
144
142
57
29
28
Normal Mode (cm$^{-1}$)
0 2 4 6 8 10
Time (ps)
Variance
IHB$^{OH}$
IHB$^{OH}$
IHB$^{OH}$
Bend$^{HOH}$
Bend$^{HOH}$
Bend$^{HOH}$
Bend$^{OOP}$
Bend$^{OOP}$
Bend$^{OOP}$
Rock
Rock
Rock

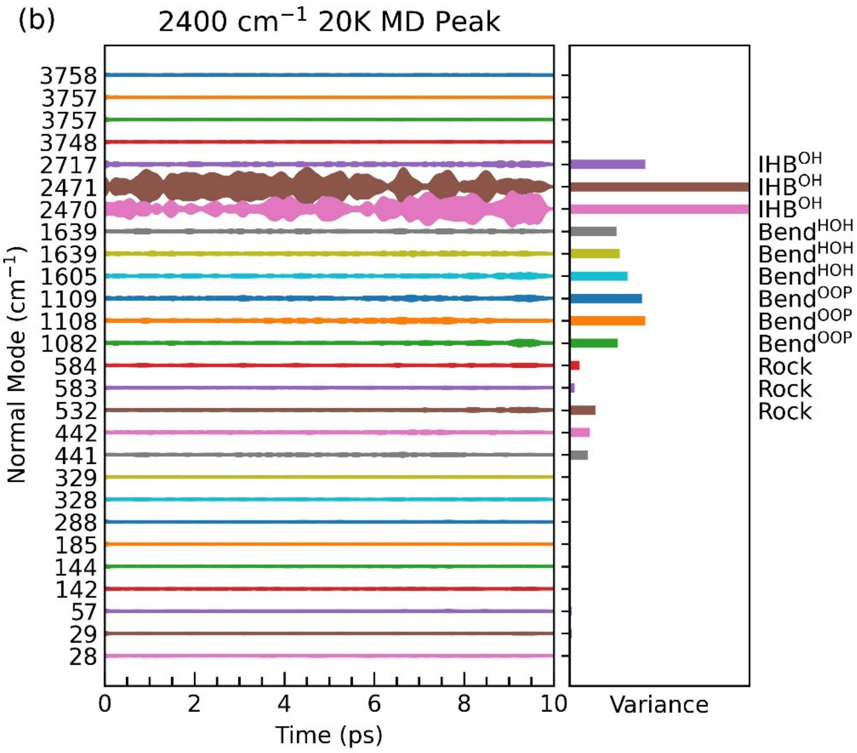
(b)
2400 cm$^{-1}$ 20K MD Peak
3758
3757
3757
3748
2717
2471
2470
1639
1639
1605
1109
1108
1082
584
583
532
442
441
329
328
288
185
144
142
57
29
28
Normal Mode (cm$^{-1}$)
0 2 4 6 8 10
Time (ps)
Variance
IHB$^{OH}$
IHB$^{OH}$
IHB$^{OH}$
Bend$^{HOH}$
Bend$^{HOH}$
Bend$^{HOH}$
Bend$^{OOP}$
Bend$^{OOP}$
Bend$^{OOP}$
Rock
Rock
Rock

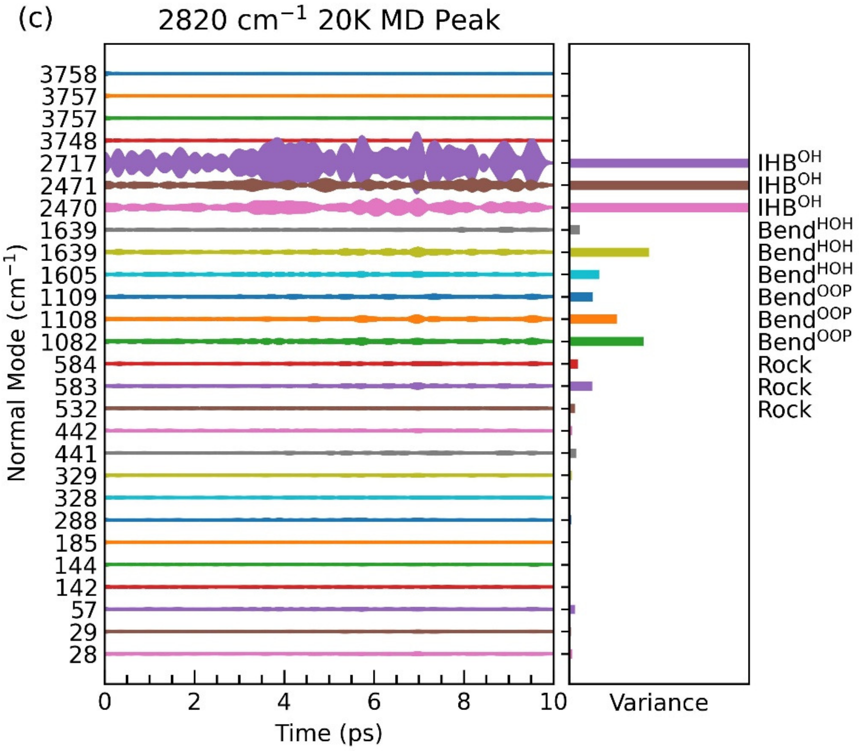


**Figure S6** Positional projection amplitudes of a reconstructed PBE0 CNEO-MD trajectory at 20K onto harmonic normal modes, along with the variance of each projection amplitude over 10 ps, for the (a) 2175 cm$^{-1}$, (b) 2400 cm$^{-1}$ peaks and (c) 2820 cm$^{-1}$ peaks of $OH^{-}\cdot(H_2O)_3$. To highlight the contributions of less dominant modes, the variances of the strongest contributors, which are typically nearby fundamental modes that the peak resonates with, are capped at the right edge and not shown at full scale.

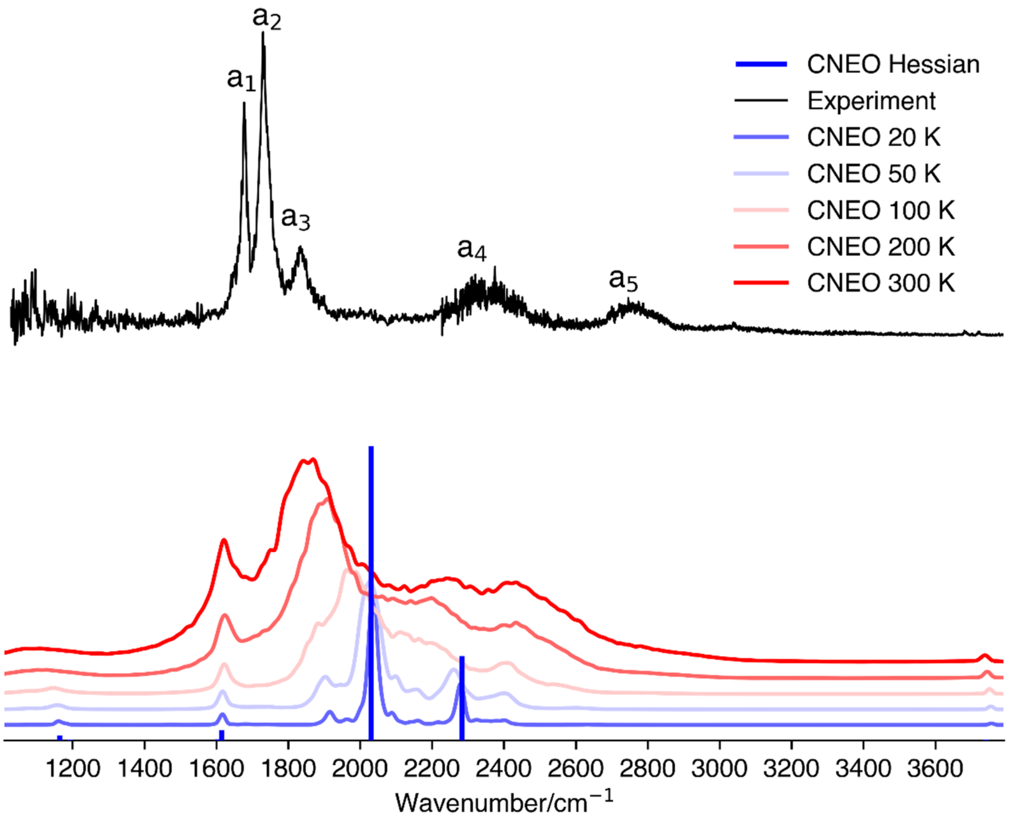


**Figure S7** IR spectra of $OH^-\cdot(H_2O)_2$ obtained from CNEO-DFT harmonic analysis (blue bars) and CNEO-MD ωB97MV simulations at different temperatures (20 K, 50 K, 100 K, 200 K, and 300 K), compared with the experimental vibrational predissociation spectrum of the $H_2$-tagged $OH^-\cdot(H_2O)_2$ cluster.

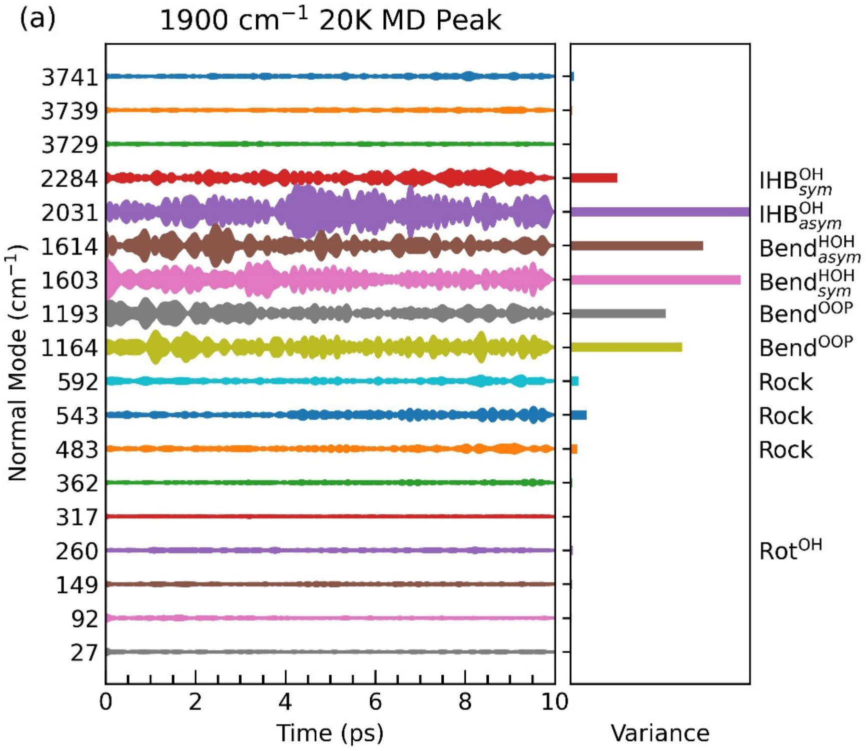
(a)
1900 cm⁻¹ 20K MD Peak
Normal Mode (cm⁻¹)
3741
3739
3729
2284
2031
1614
1603
1193
1164
592
543
483
362
317
260
149
92
27
0
2
4
6
8
10
Time (ps)
Variance
IHB$^{OH}_{sym}$
IHB$^{OH}_{asym}$
Bend$^{HOH}_{asym}$
Bend$^{HOH}_{sym}$
Bend$^{OOP}$
Bend$^{OOP}$
Rock
Rock
Rock
Rot$^{OH}$

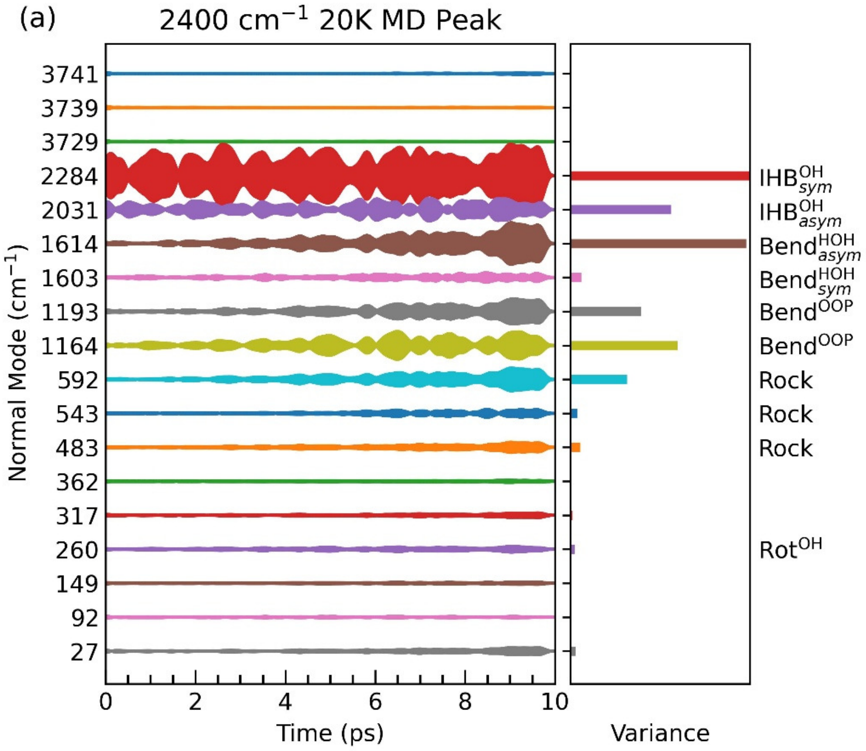
(a)
2400 cm⁻¹ 20K MD Peak
Normal Mode (cm⁻¹)
3741
3739
3729
2284
2031
1614
1603
1193
1164
592
543
483
362
317
260
149
92
27
0
2
4
6
8
10
Time (ps)
Variance
IHB$^{OH}_{sym}$
IHB$^{OH}_{asym}$
Bend$^{HOH}_{asym}$
Bend$^{HOH}_{sym}$
Bend$^{OOP}$
Bend$^{OOP}$
Rock
Rock
Rock
Rot$^{OH}$

**Figure S8** Positional projection amplitudes of a reconstructed ωB97MV CNEO-MD trajectory at 20K onto harmonic normal modes, along with the variance of each projection amplitude over 10 ps, for the (a) 1900 cm$^{-1}$ and (b) 2400 cm$^{-1}$ peaks of $OH^{-}\cdot(H_2O)_2$. To highlight the contributions of less dominant modes, the variances of the strongest contributors, which are typically nearby fundamental modes that the peak resonates with, are capped at the right edge and not shown at full scale.

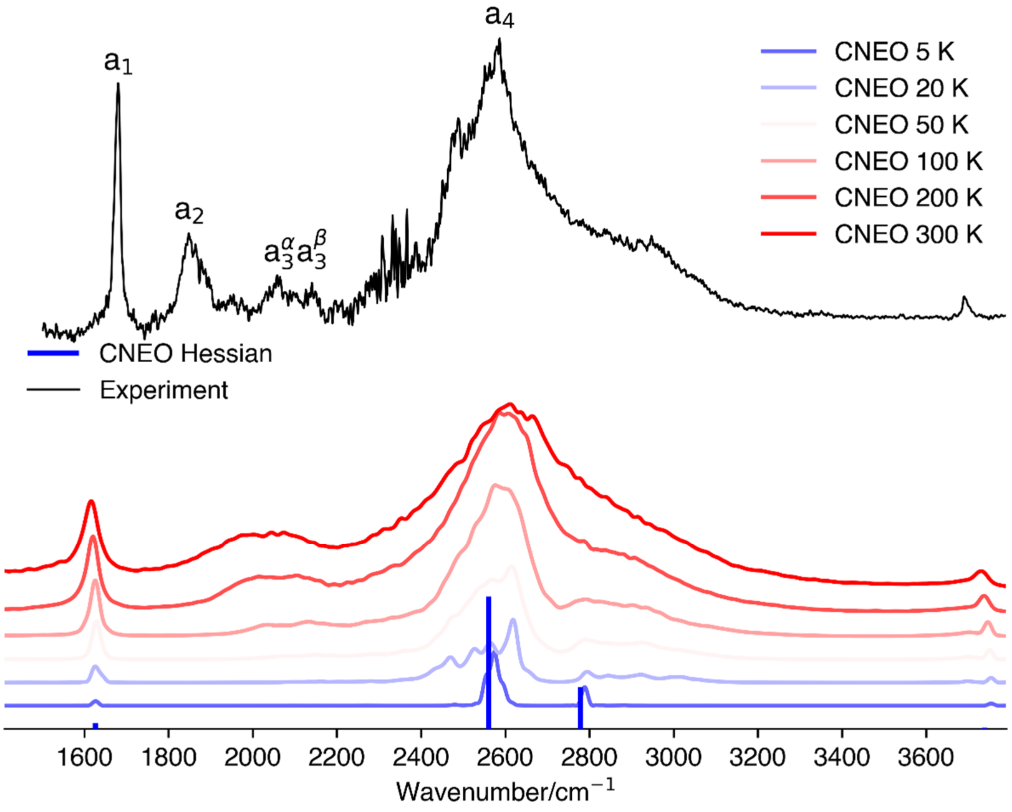


**Figure S9** IR spectra of $OH^{-}\cdot(H_2O)_3$ obtained from CNEO-DFT harmonic analysis (blue bars) and CNEO-MD ωB97MV simulations at different temperatures (20 K, 50 K, 100 K, 200 K, and 300 K), compared with the experimental vibrational predissociation spectrum of the $H_2$-tagged $OH^{-}\cdot(H_2O)_3$ cluster.

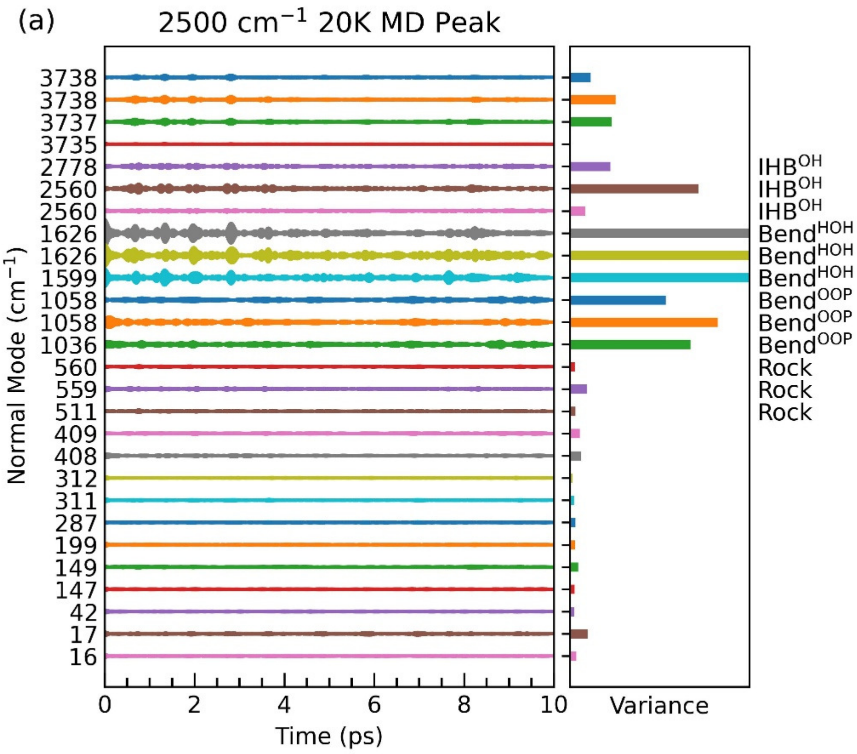
(a)
2500 cm$^{-1}$ 20K MD Peak
Normal Mode (cm$^{-1}$)
3738
3738
3737
3735
2778
2560
2560
1626
1626
1599
1058
1058
1036
560
559
511
409
408
312
311
287
199
149
147
42
17
16
IHB$^{OH}$
IHB$^{OH}$
IHB$^{OH}$
Bend$^{HOH}$
Bend$^{HOH}$
Bend$^{HOH}$
Bend$^{OOP}$
Bend$^{OOP}$
Bend$^{OOP}$
Rock
Rock
Rock
0
2
4
6
8
10
Time (ps)
Variance

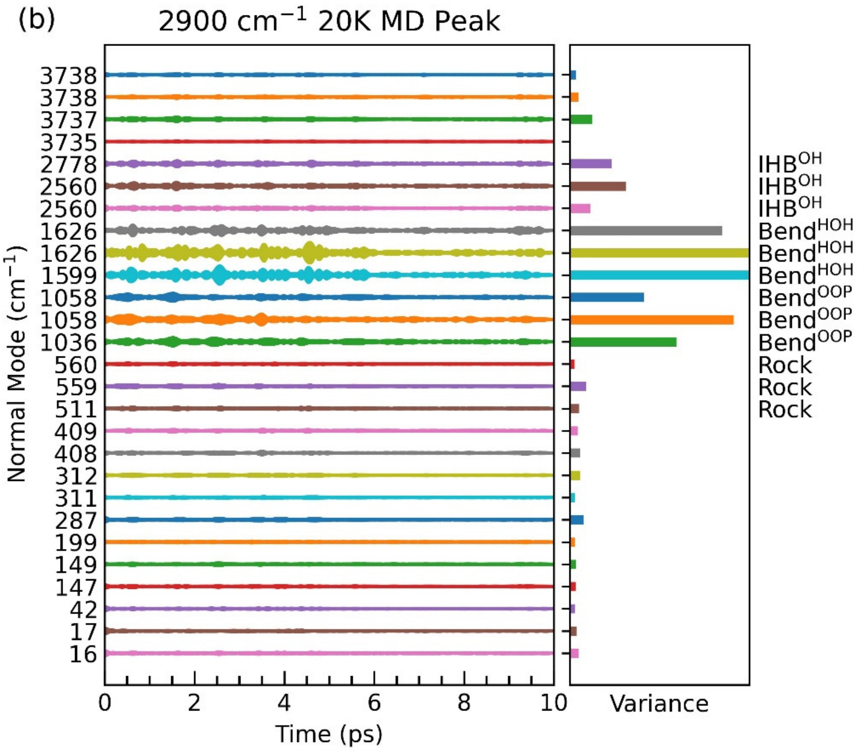
(b)
2900 cm$^{-1}$ 20K MD Peak
Normal Mode (cm$^{-1}$)
3738
3738
3737
3735
2778
2560
2560
1626
1626
1599
1058
1058
1036
560
559
511
409
408
312
311
287
199
149
147
42
17
16
IHB$^{OH}$
IHB$^{OH}$
IHB$^{OH}$
Bend$^{HOH}$
Bend$^{HOH}$
Bend$^{HOH}$
Bend$^{OOP}$
Bend$^{OOP}$
Bend$^{OOP}$
Rock
Rock
Rock
0
2
4
6
8
10
Time (ps)
Variance

**Figure S10** Positional projection amplitudes of a reconstructed ωB97MV CNEO-MD trajectory at 20K onto harmonic normal modes, along with the variance of each projection amplitude over 10 ps, for the (a) 2500 $cm^{-1}$ and (b) 2900 $cm^{-1}$ peaks of $OH^{-}\cdot(H_2O)_3$. To highlight the contributions of less dominant modes, the variances of the strongest contributors, which are typically nearby fundamental modes that the peak resonates with, are capped at the right edge and not shown at full scale.

## Reference


(1) Wang, H.; Zhang, L.; Han, J.; E, W. DeePMD-Kit: A Deep Learning Package for Many-Body Potential Energy Representation and Molecular Dynamics. *Computer Physics Communications* **2018**, *228*, 178–184. https://doi.org/10.1016/j.cpc.2018.03.016.

(2) Zeng, J.; Zhang, D.; Lu, D.; Mo, P.; Li, Z.; Chen, Y.; Rynik, M.; Huang, L.; Li, Z.; Shi, S.; Wang, Y.; Ye, H.; Tuo, P.; Yang, J.; Ding, Y.; Li, Y.; Tisi, D.; Zeng, Q.; Bao, H.; Xia, Y.; Huang, J.; Muraoka, K.; Wang, Y.; Chang, J.; Yuan, F.; Bore, S. L.; Cai, C.; Lin, Y.; Wang, B.; Xu, J.; Zhu, J.-X.; Luo, C.; Zhang, Y.; Goodall, R. E. A.; Liang, W.; Singh, A. K.; Yao, S.; Zhang, J.; Wentzcovitch, R.; Han, J.; Liu, J.; Jia, W.; York, D. M.; E, W.; Car, R.; Zhang, L.; Wang, H. DeePMD-Kit v2: A Software Package for Deep Potential Models. *The Journal of Chemical Physics* **2023**, *159* (5), 054801. https://doi.org/10.1063/5.0155600.

(3) Zhang, L.; Han, J.; Wang, H.; Saidi, W.; Car, R.; E, W. End-to-End Symmetry Preserving Inter-Atomic Potential Energy Model for Finite and Extended Systems. In *Advances in Neural Information Processing Systems*; Curran Associates, Inc., 2018; Vol. 31.
