# Supplementary figures and images for: Characterizing Infrared Spectra of OH–·(H2O)2 and OH–·(H2O)3 with Constrained Nuclear-Electronic Orbital Molecular Dynamics
Source: J Phys Chem A. 2025 Oct 18;129(43):9883–94. doi: 10.1021/acs.jpca.5c04334 (PMC12581138; doi:10.1021/acs.jpca.5c04334)

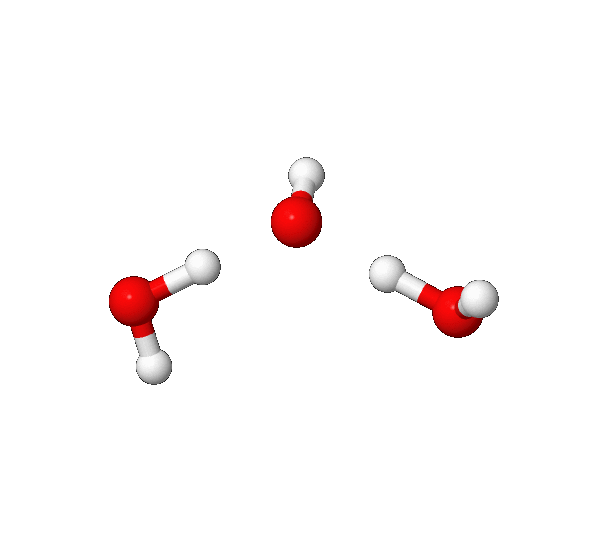

Supplement: Supplementary file 2 [file jp5c04334_si_002.zip › vibrations/2H2O/vibration_10.gif]

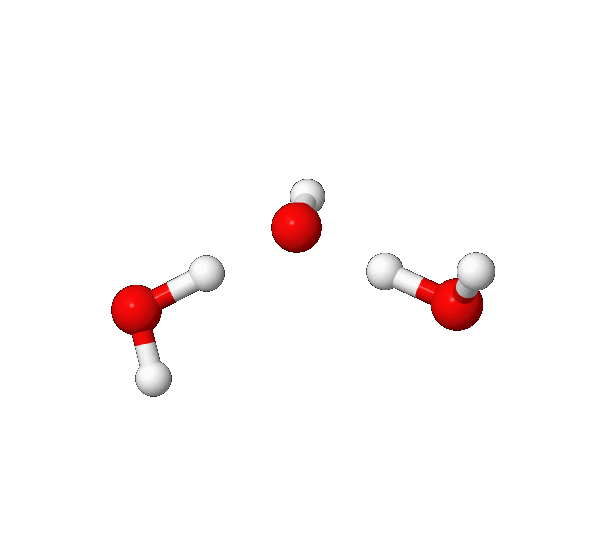

Supplement: Supplementary file 2 [file jp5c04334_si_002.zip › vibrations/2H2O/vibration_11.gif]

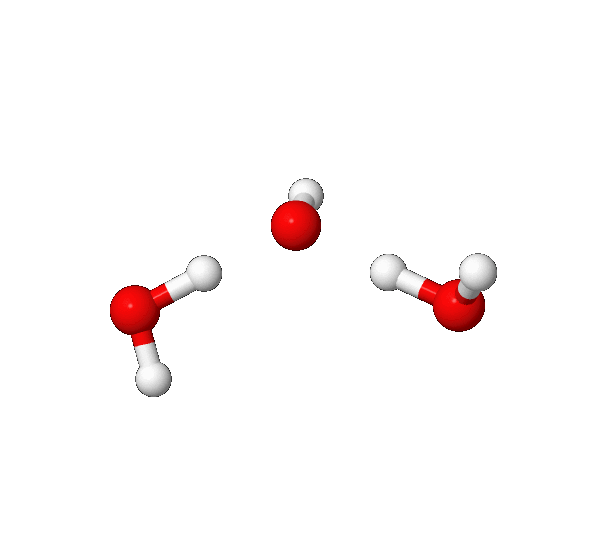

Supplement: Supplementary file 2 [file jp5c04334_si_002.zip › vibrations/2H2O/vibration_12.gif]

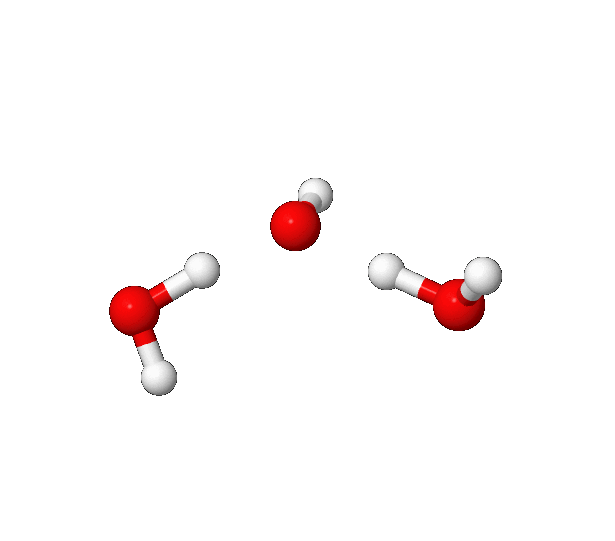

Supplement: Supplementary file 2 [file jp5c04334_si_002.zip › vibrations/2H2O/vibration_13.gif]

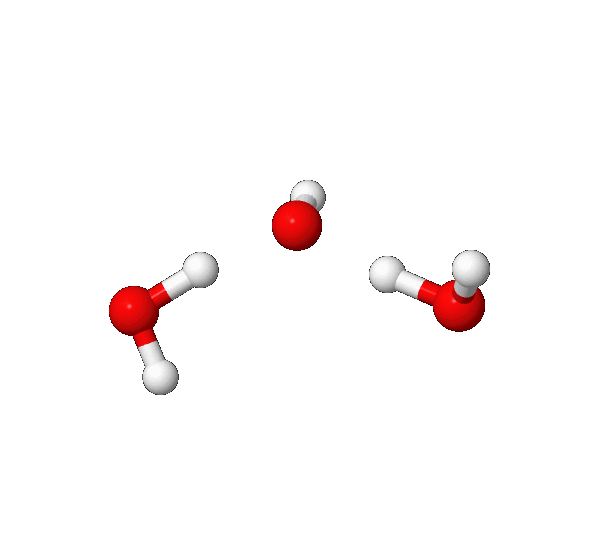

Supplement: Supplementary file 2 [file jp5c04334_si_002.zip › vibrations/2H2O/vibration_14.gif]

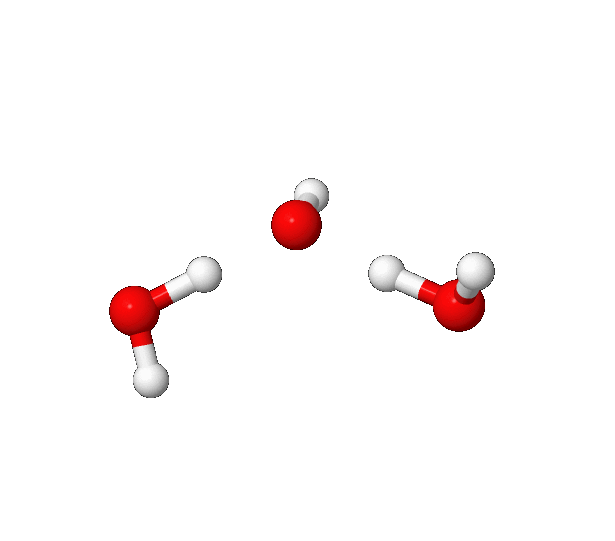

Supplement: Supplementary file 2 [file jp5c04334_si_002.zip › vibrations/2H2O/vibration_15.gif]

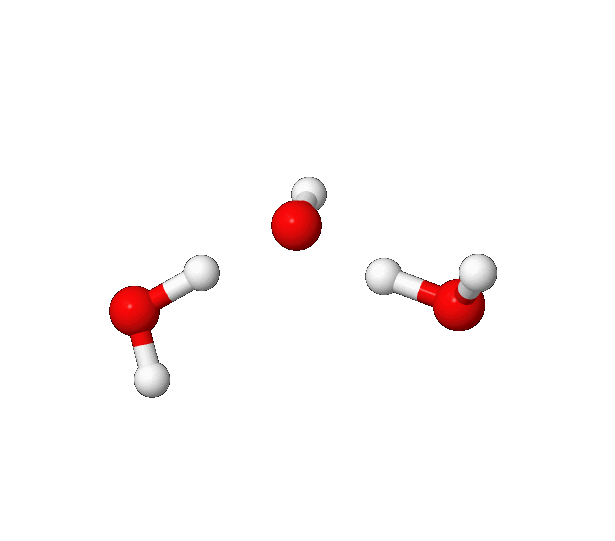

Supplement: Supplementary file 2 [file jp5c04334_si_002.zip › vibrations/2H2O/vibration_16.gif]

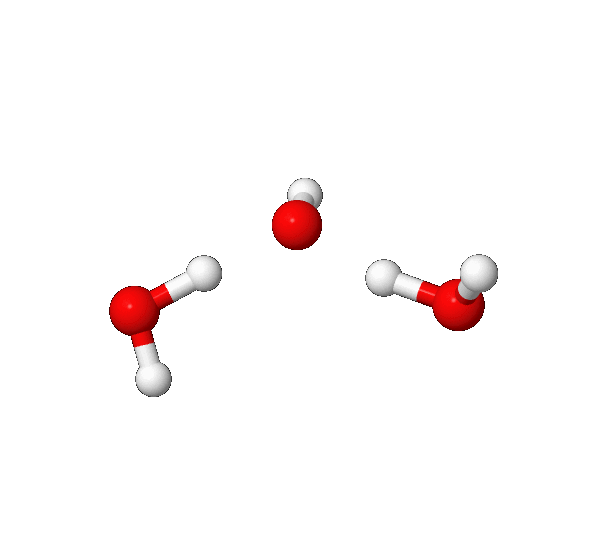

Supplement: Supplementary file 2 [file jp5c04334_si_002.zip › vibrations/2H2O/vibration_17.gif]

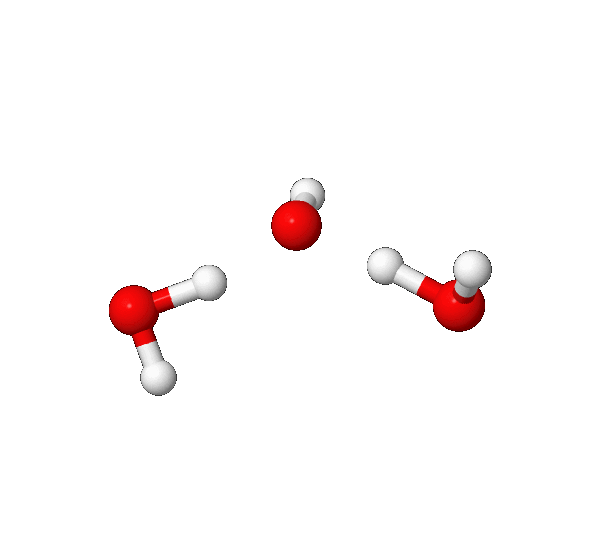

Supplement: Supplementary file 2 [file jp5c04334_si_002.zip › vibrations/2H2O/vibration_18.gif]

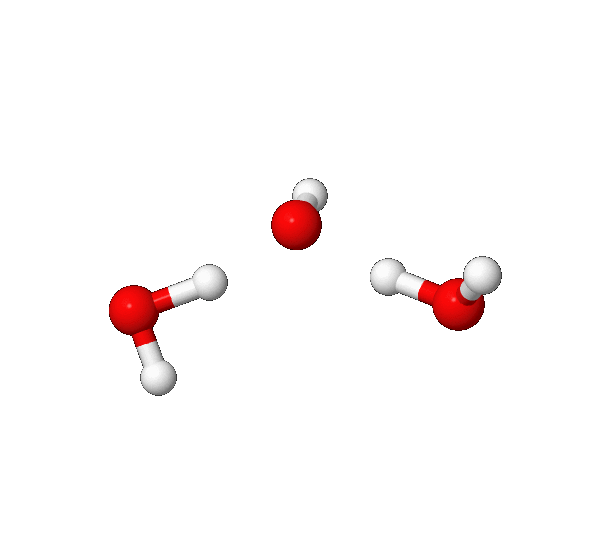

Supplement: Supplementary file 2 [file jp5c04334_si_002.zip › vibrations/2H2O/vibration_19.gif]

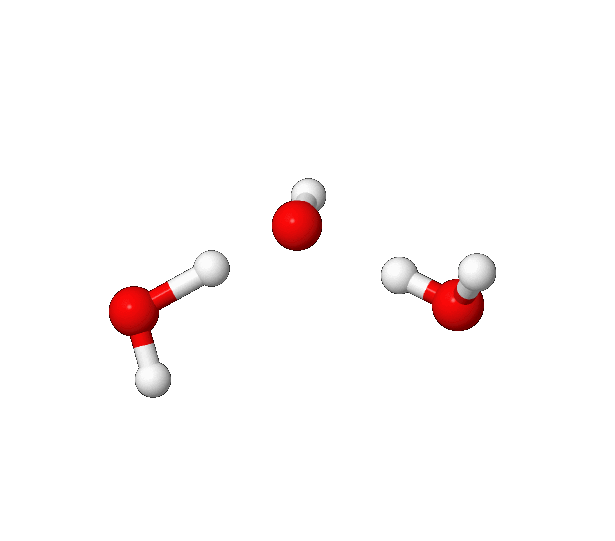

Supplement: Supplementary file 2 [file jp5c04334_si_002.zip › vibrations/2H2O/vibration_20.gif]

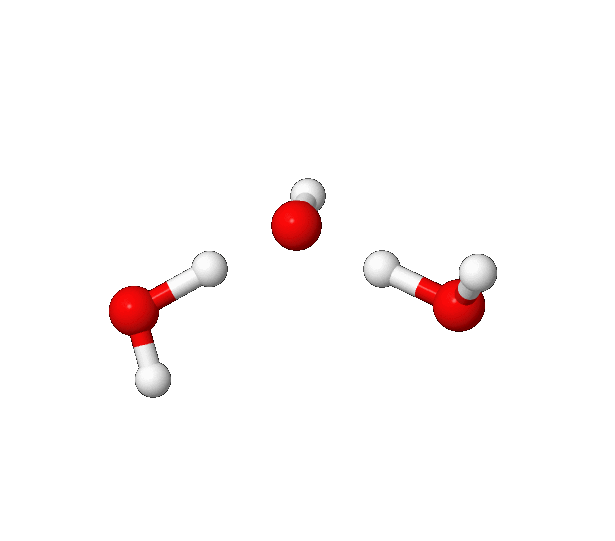

Supplement: Supplementary file 2 [file jp5c04334_si_002.zip › vibrations/2H2O/vibration_21.gif]

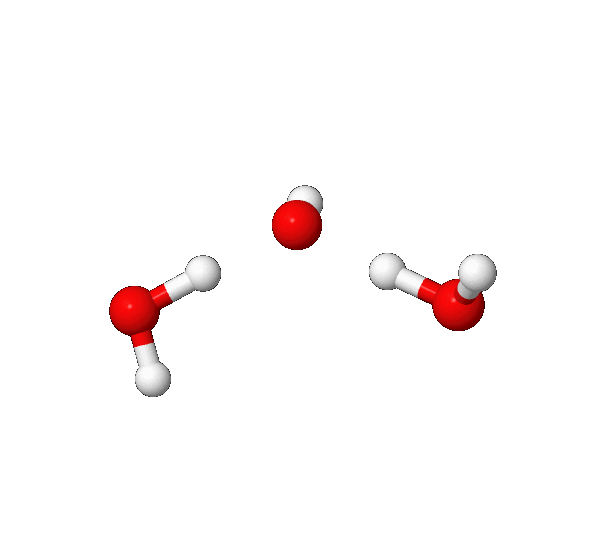

Supplement: Supplementary file 2 [file jp5c04334_si_002.zip › vibrations/2H2O/vibration_22.gif]

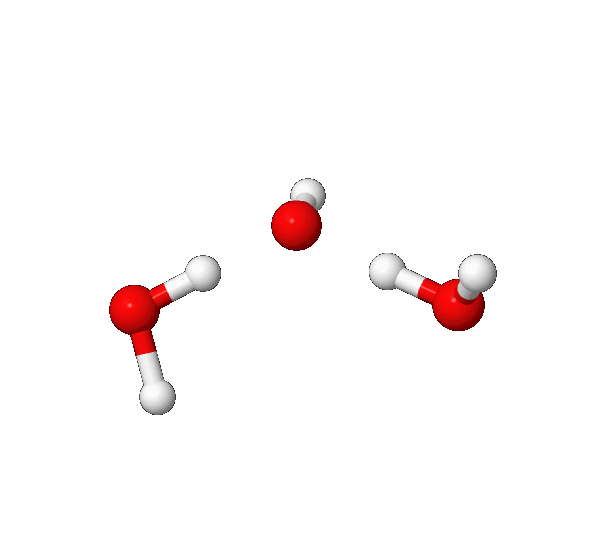

Supplement: Supplementary file 2 [file jp5c04334_si_002.zip › vibrations/2H2O/vibration_23.gif]

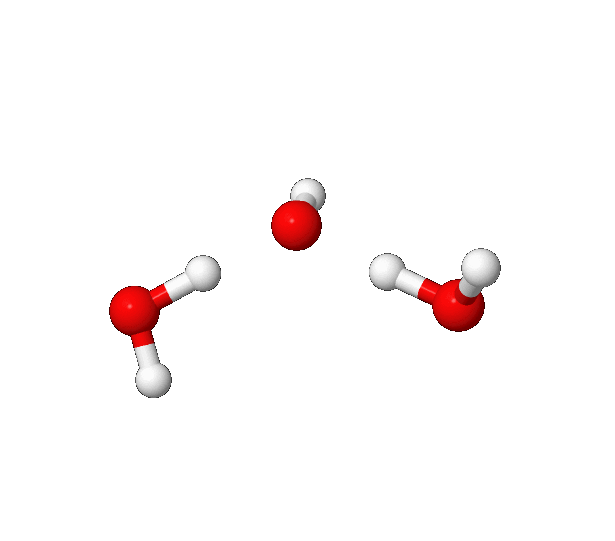

Supplement: Supplementary file 2 [file jp5c04334_si_002.zip › vibrations/2H2O/vibration_24.gif]

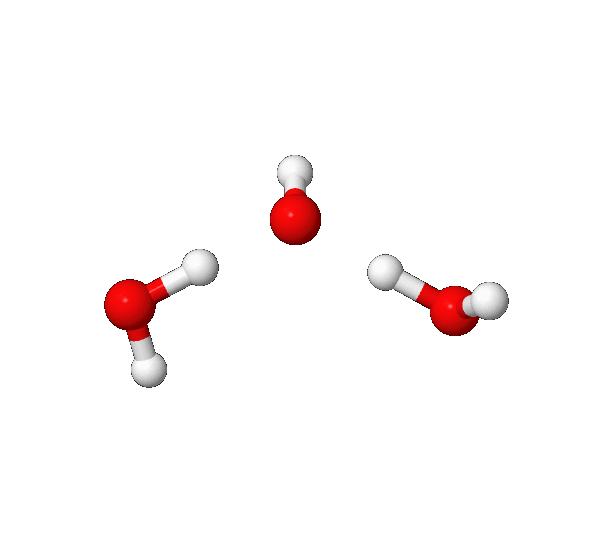

Supplement: Supplementary file 2 [file jp5c04334_si_002.zip › vibrations/2H2O/vibration_7.gif]

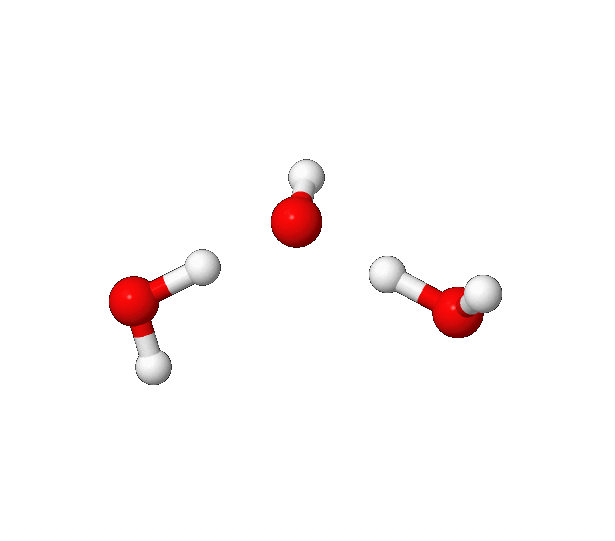

Supplement: Supplementary file 2 [file jp5c04334_si_002.zip › vibrations/2H2O/vibration_8.gif]

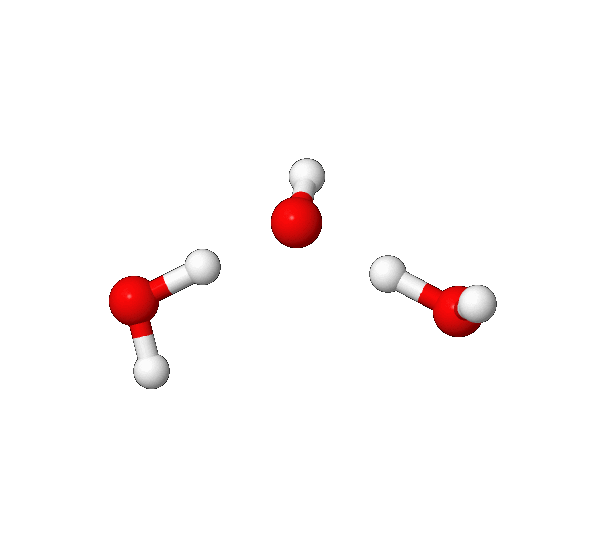

Supplement: Supplementary file 2 [file jp5c04334_si_002.zip › vibrations/2H2O/vibration_9.gif]

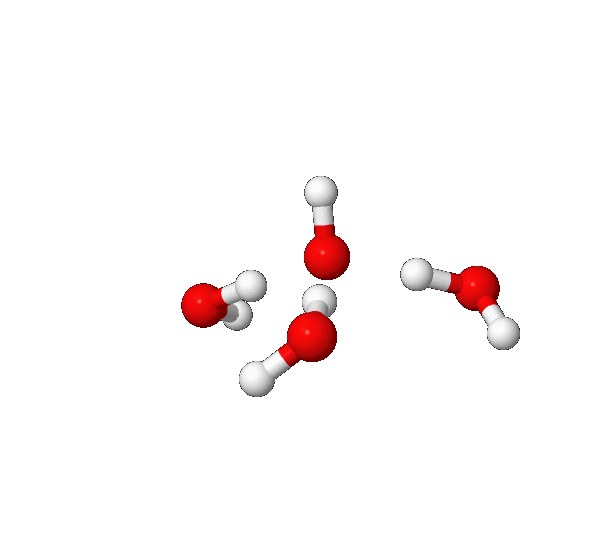

Supplement: Supplementary file 2 [file jp5c04334_si_002.zip › vibrations/3H2O/vibration_10.gif]

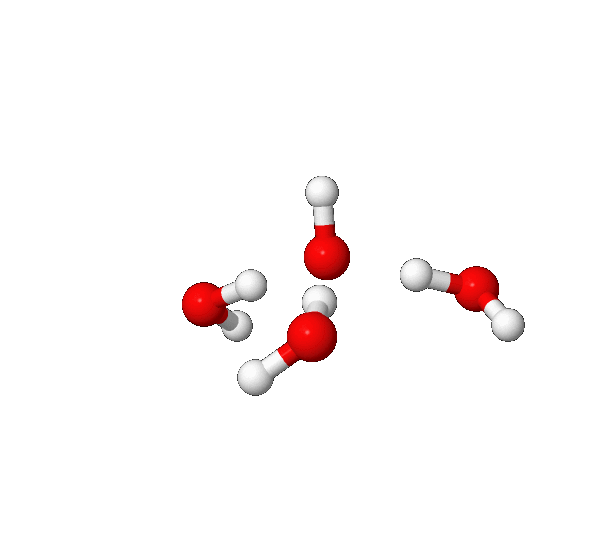

Supplement: Supplementary file 2 [file jp5c04334_si_002.zip › vibrations/3H2O/vibration_11.gif]

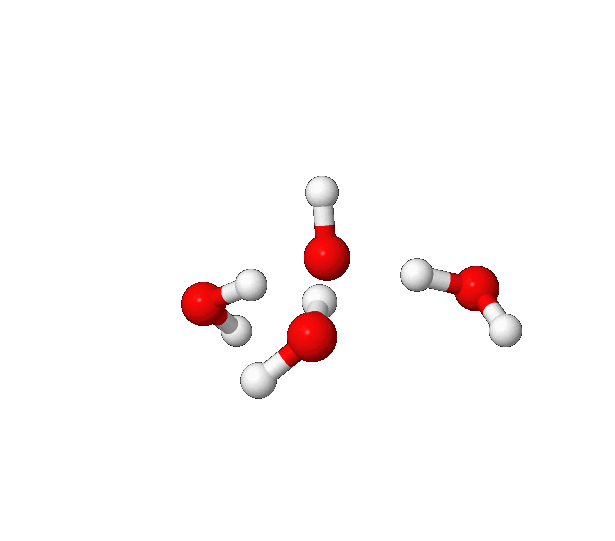

Supplement: Supplementary file 2 [file jp5c04334_si_002.zip › vibrations/3H2O/vibration_12.gif]

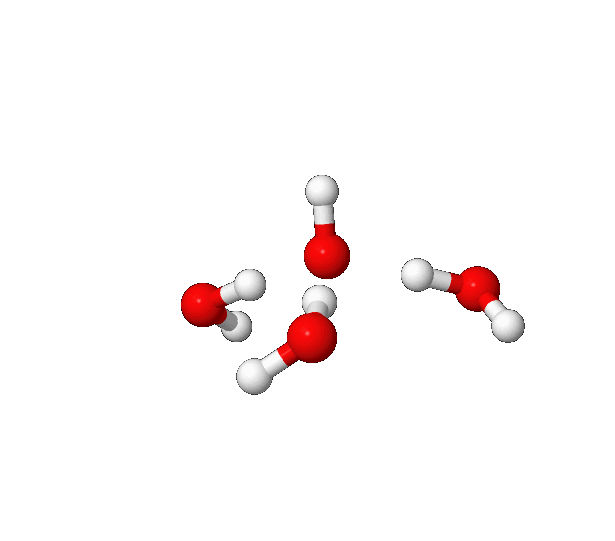

Supplement: Supplementary file 2 [file jp5c04334_si_002.zip › vibrations/3H2O/vibration_13.gif]

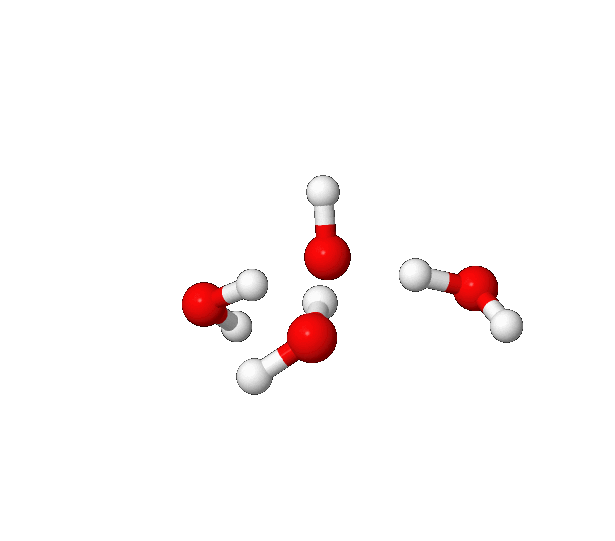

Supplement: Supplementary file 2 [file jp5c04334_si_002.zip › vibrations/3H2O/vibration_14.gif]

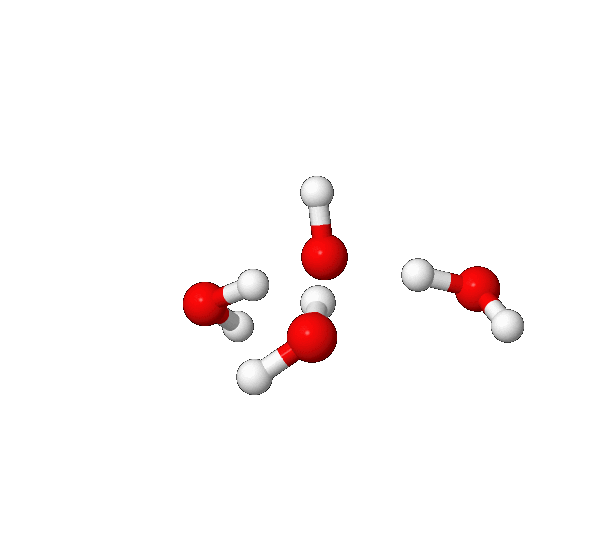

Supplement: Supplementary file 2 [file jp5c04334_si_002.zip › vibrations/3H2O/vibration_15.gif]

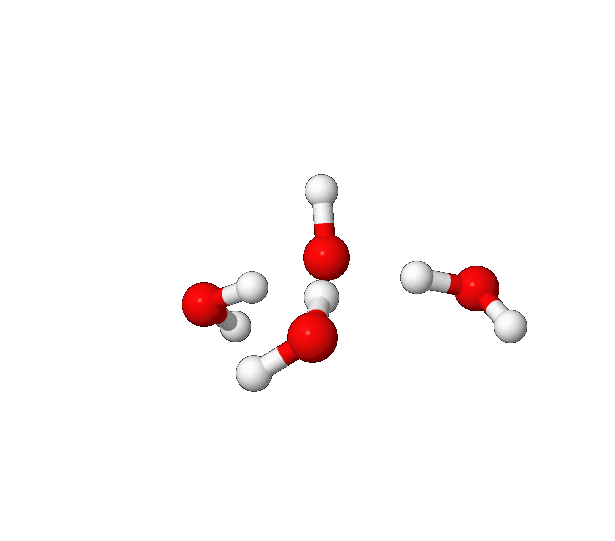

Supplement: Supplementary file 2 [file jp5c04334_si_002.zip › vibrations/3H2O/vibration_16.gif]

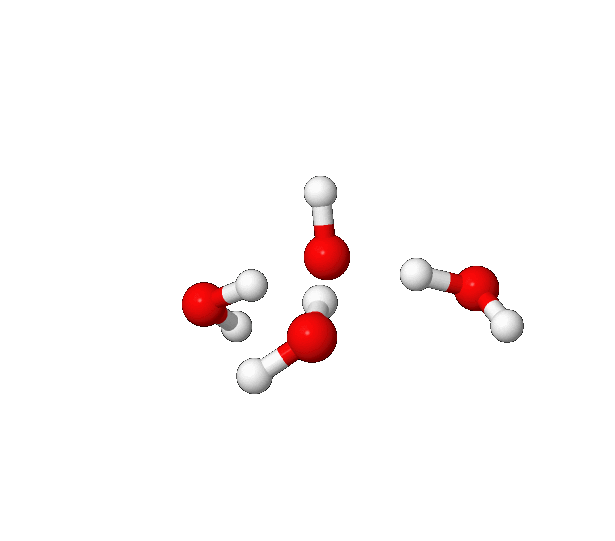

Supplement: Supplementary file 2 [file jp5c04334_si_002.zip › vibrations/3H2O/vibration_17.gif]

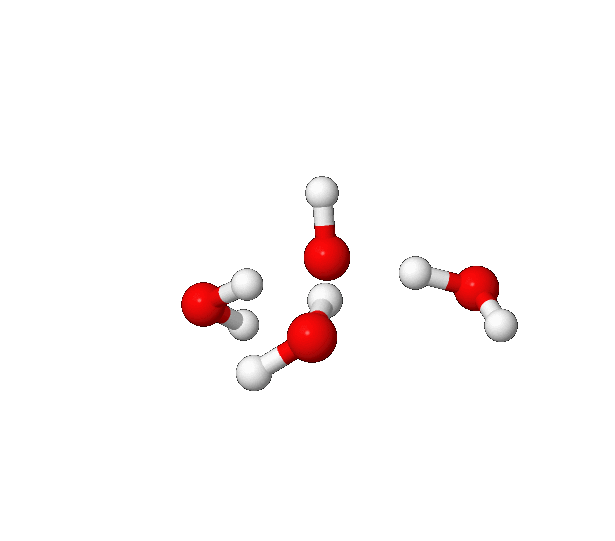

Supplement: Supplementary file 2 [file jp5c04334_si_002.zip › vibrations/3H2O/vibration_18.gif]

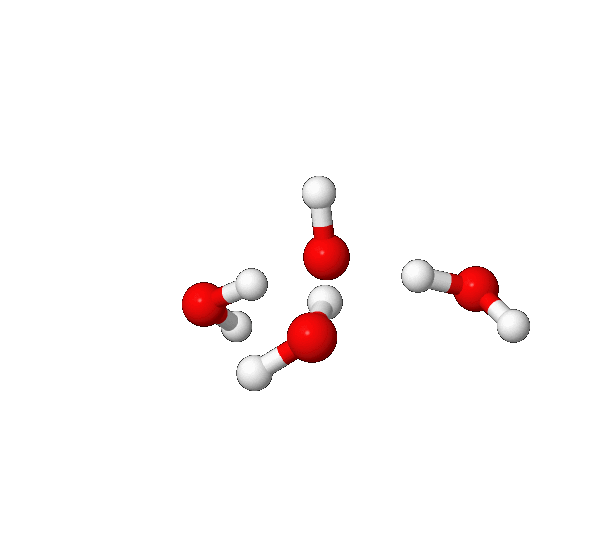

Supplement: Supplementary file 2 [file jp5c04334_si_002.zip › vibrations/3H2O/vibration_19.gif]

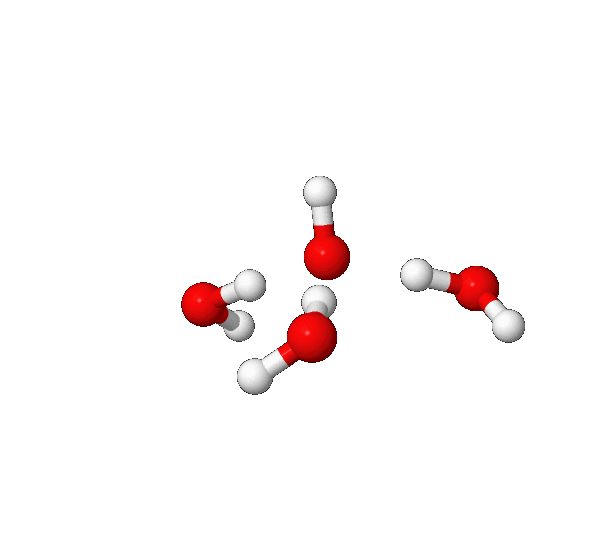

Supplement: Supplementary file 2 [file jp5c04334_si_002.zip › vibrations/3H2O/vibration_20.gif]

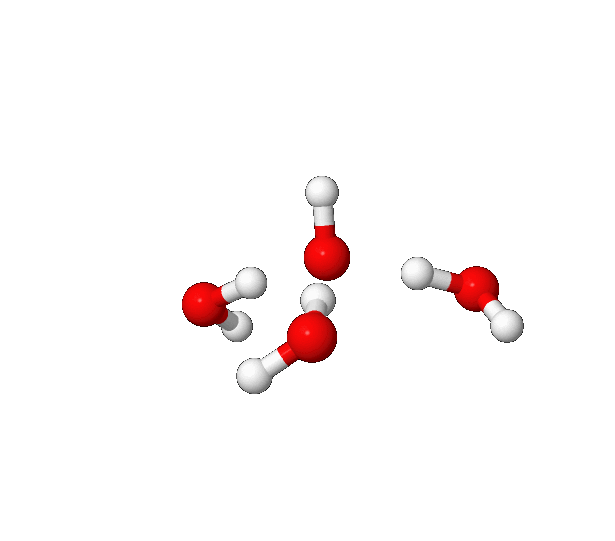

Supplement: Supplementary file 2 [file jp5c04334_si_002.zip › vibrations/3H2O/vibration_21.gif]

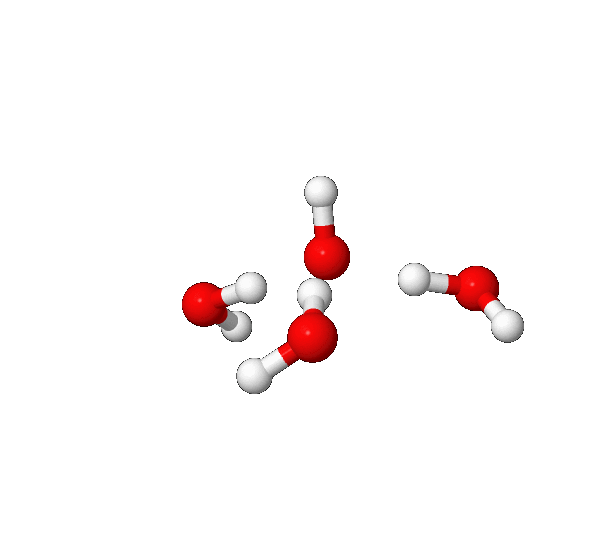

Supplement: Supplementary file 2 [file jp5c04334_si_002.zip › vibrations/3H2O/vibration_22.gif]

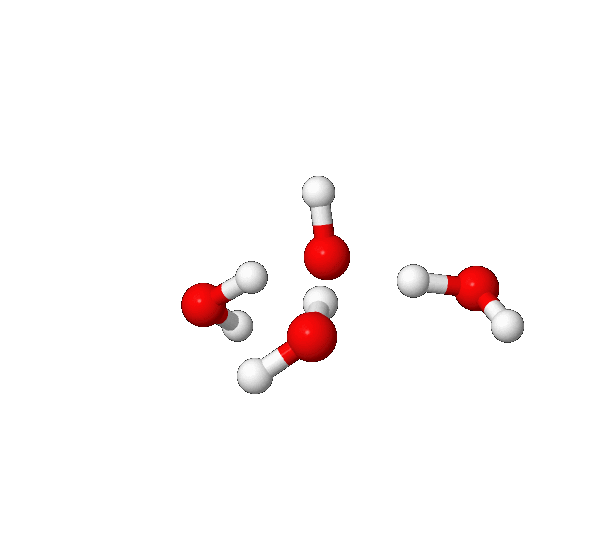

Supplement: Supplementary file 2 [file jp5c04334_si_002.zip › vibrations/3H2O/vibration_23.gif]

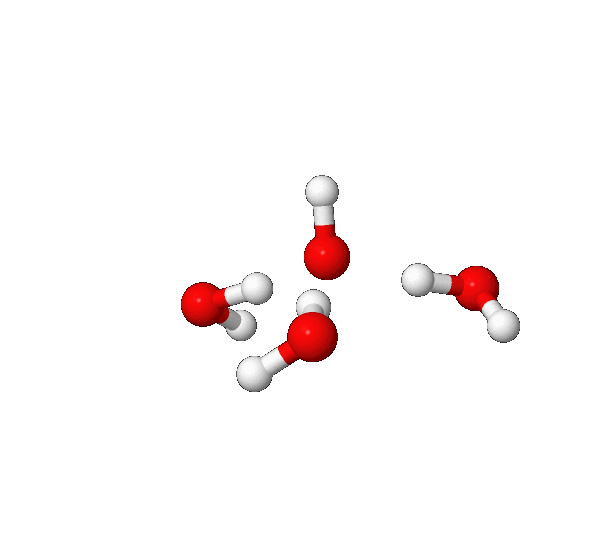

Supplement: Supplementary file 2 [file jp5c04334_si_002.zip › vibrations/3H2O/vibration_24.gif]

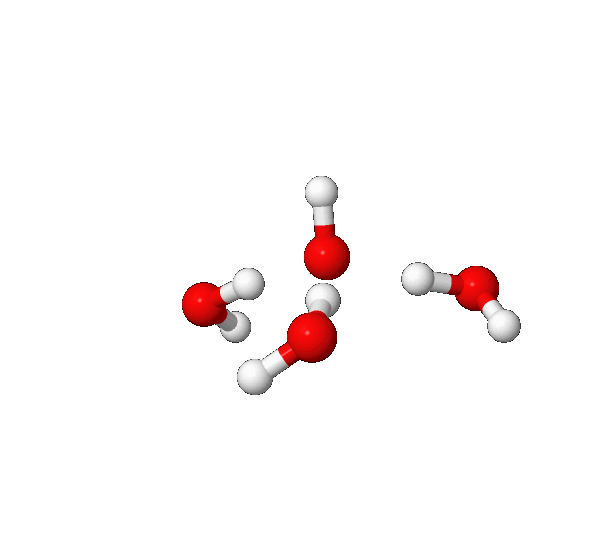

Supplement: Supplementary file 2 [file jp5c04334_si_002.zip › vibrations/3H2O/vibration_25.gif]

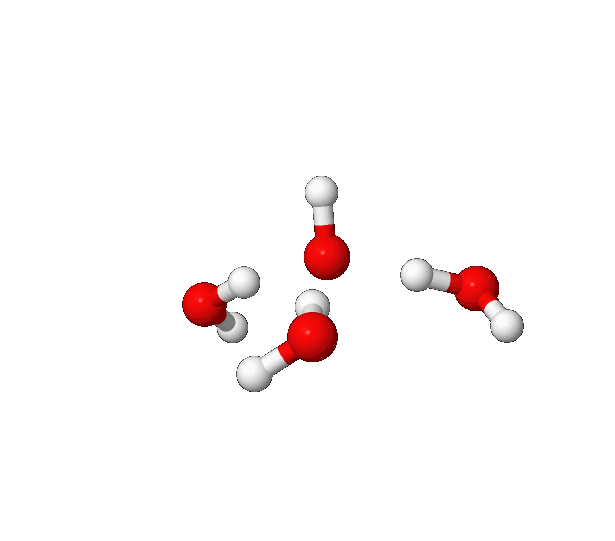

Supplement: Supplementary file 2 [file jp5c04334_si_002.zip › vibrations/3H2O/vibration_26.gif]

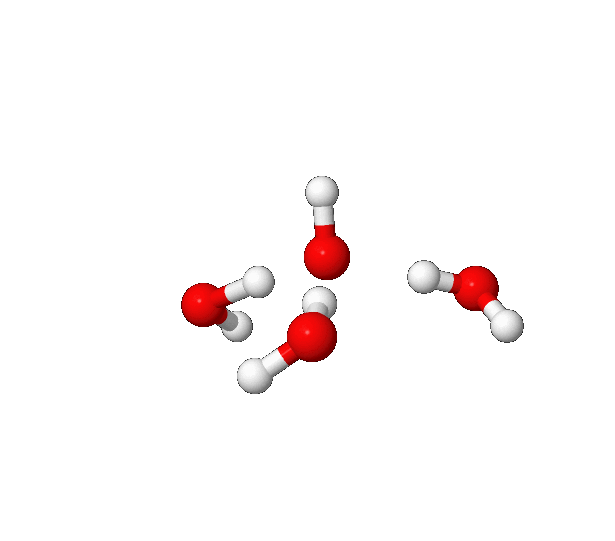

Supplement: Supplementary file 2 [file jp5c04334_si_002.zip › vibrations/3H2O/vibration_27.gif]

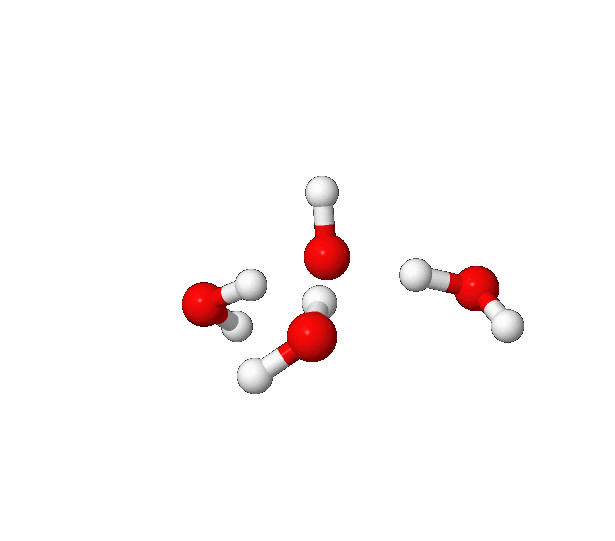

Supplement: Supplementary file 2 [file jp5c04334_si_002.zip › vibrations/3H2O/vibration_28.gif]

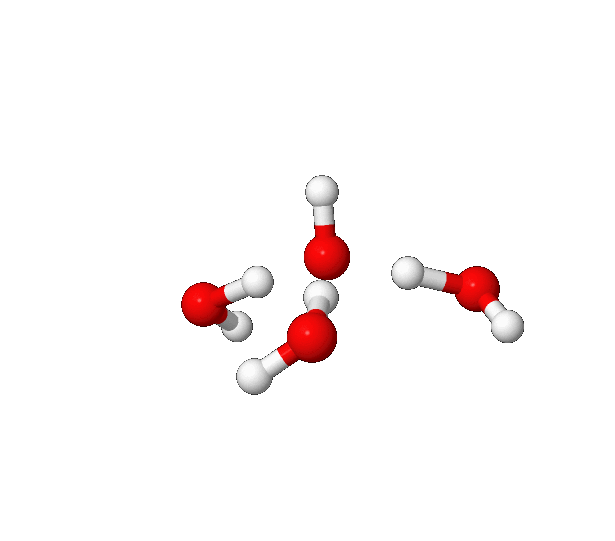

Supplement: Supplementary file 2 [file jp5c04334_si_002.zip › vibrations/3H2O/vibration_29.gif]

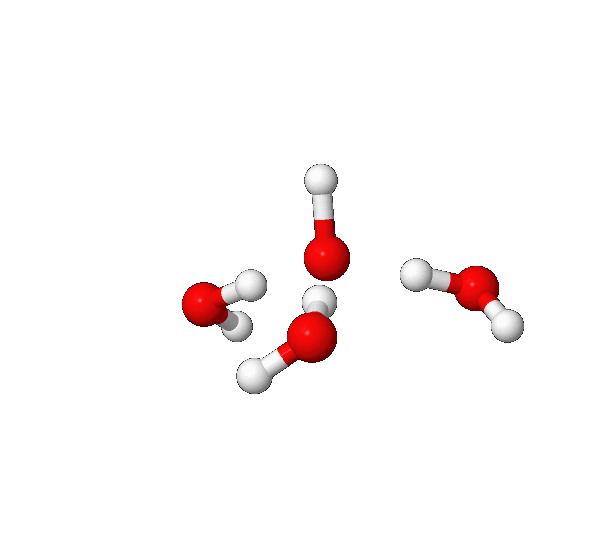

Supplement: Supplementary file 2 [file jp5c04334_si_002.zip › vibrations/3H2O/vibration_30.gif]

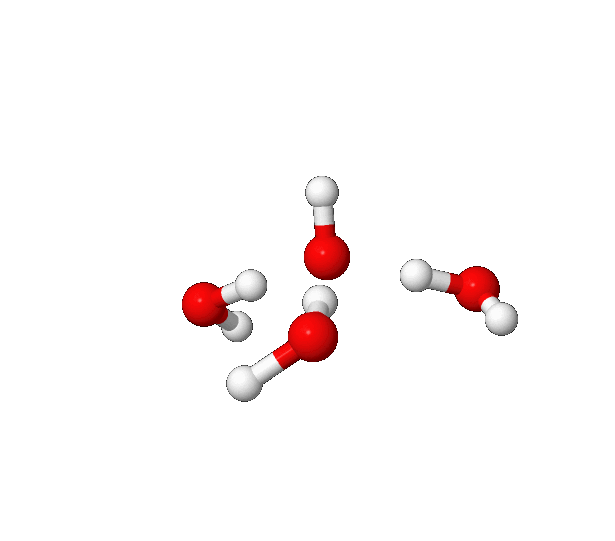

Supplement: Supplementary file 2 [file jp5c04334_si_002.zip › vibrations/3H2O/vibration_31.gif]

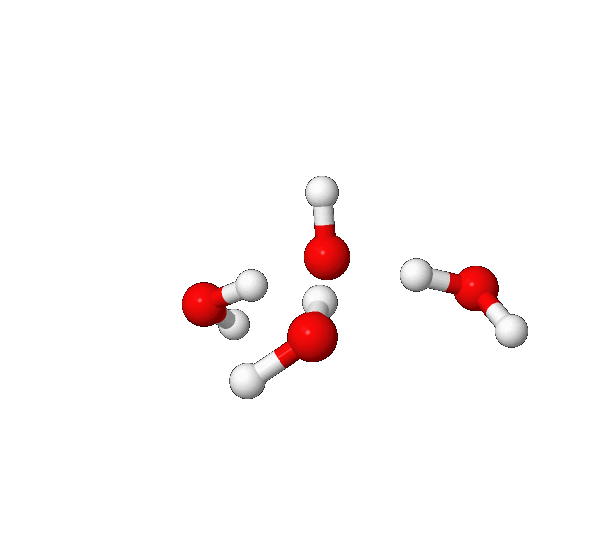

Supplement: Supplementary file 2 [file jp5c04334_si_002.zip › vibrations/3H2O/vibration_32.gif]

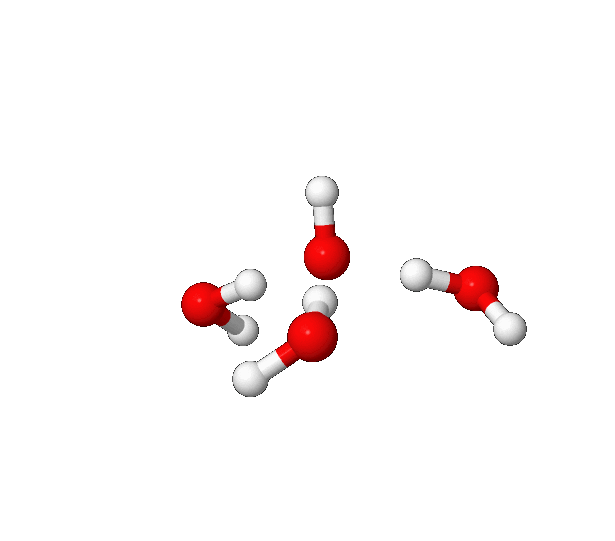

Supplement: Supplementary file 2 [file jp5c04334_si_002.zip › vibrations/3H2O/vibration_33.gif]

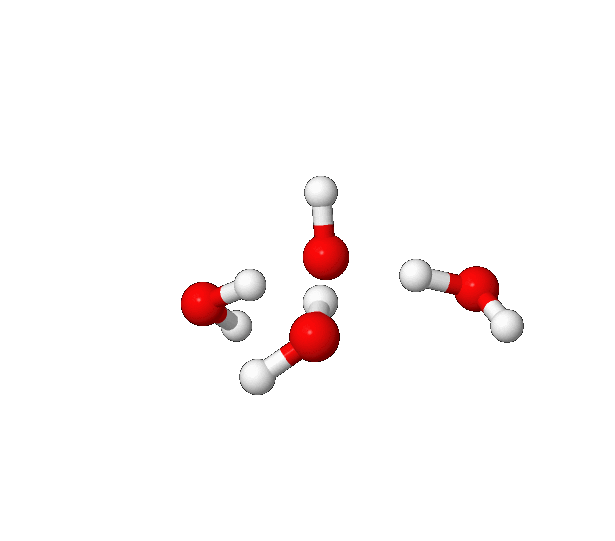

Supplement: Supplementary file 2 [file jp5c04334_si_002.zip › vibrations/3H2O/vibration_7.gif]

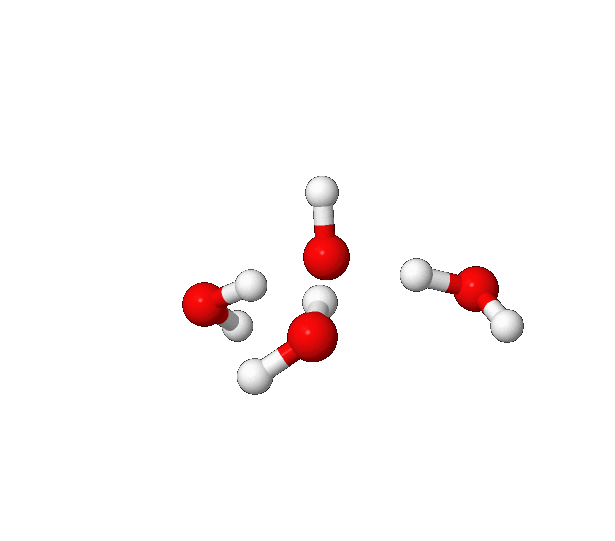

Supplement: Supplementary file 2 [file jp5c04334_si_002.zip › vibrations/3H2O/vibration_8.gif]

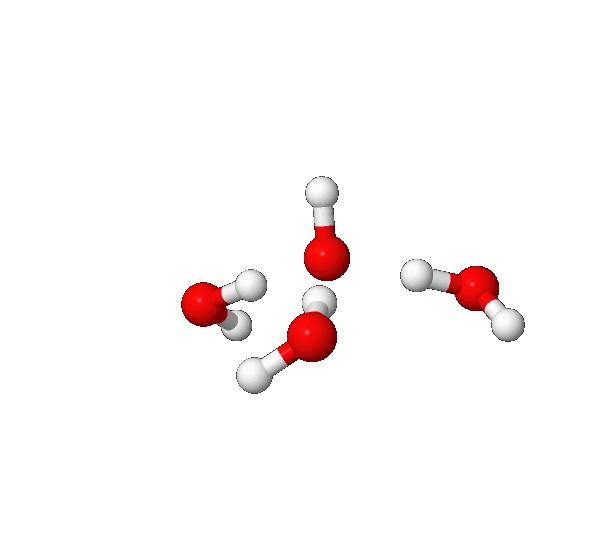

Supplement: Supplementary file 2 [file jp5c04334_si_002.zip › vibrations/3H2O/vibration_9.gif]
